# The role of anthropogenic influences on a tropical lake ecosystem and its surrounding catchment: a case study of Lake Sentani

**DOI:** 10.1093/femsec/fiae162

**Published:** 2024-12-17

**Authors:** Sulung Nomosatryo, Daniel Lipus, Alexander Bartholomäus, Cynthia Henny, Iwan Ridwansyah, Puguh Sujarta, Sizhong Yang, Dirk Wagner, Jens Kallmeyer

**Affiliations:** GFZ German Research Centre for Geosciences, Section Geomicrobiology, 14473, Potsdam, Germany; Research Center for Limnology and Water Resources, National Research and Innovation Agency (BRIN), KST Soekarno, Jalan Jakarta-Bogor KM 46, Cibinong, Bogor 16911, Indonesia; GFZ German Research Centre for Geosciences, Section Geomicrobiology, 14473, Potsdam, Germany; Department of Biological and Chemical Sciences, College of Life Sciences, Thomas Jefferson University, Philadelphia, PA 19144, United States; GFZ German Research Centre for Geosciences, Section Geomicrobiology, 14473, Potsdam, Germany; Research Center for Limnology and Water Resources, National Research and Innovation Agency (BRIN), KST Soekarno, Jalan Jakarta-Bogor KM 46, Cibinong, Bogor 16911, Indonesia; Research Center for Limnology and Water Resources, National Research and Innovation Agency (BRIN), KST Soekarno, Jalan Jakarta-Bogor KM 46, Cibinong, Bogor 16911, Indonesia; Cendrawasih University, Department of Biology, Faculty of Mathematics and Natural Sciences, Jl. Kamp. Wolker, Waena, Jayapura 99358, Indonesia; GFZ German Research Centre for Geosciences, Section Geomicrobiology, 14473, Potsdam, Germany; GFZ German Research Centre for Geosciences, Section Geomicrobiology, 14473, Potsdam, Germany; University of Potsdam, Institute of Geosciences, 14476, Potsdam, Germany; GFZ German Research Centre for Geosciences, Section Geomicrobiology, 14473, Potsdam, Germany

**Keywords:** microbial communities, tropical lake, limnology, surface sediment

## Abstract

Lake Sentani is a tropical lake in Indonesia, consisting of four interconnected sub-basins of different water depths. While previous work has highlighted the impact of catchment composition on biogeochemical processes in Lake Sentani, little is currently known about the microbiological characteristics across this unique ecosystem. With recent population growth in this historically rural area, the anthropogenic impact on Lake Sentani and hence its microbial life is also increasing. Therefore, we aimed to explore the influence of environmental and anthropogenic factors on the microbial diversity of Lake Sentani. Here, we present a detailed microbiological evaluation of Lake Sentani, analyzing 49 different sites across the lake, its tributary rivers and their river mouths to assess diversity and community structure using 16S rRNA gene sequencing. Our results reveal distinct communities in lake and river sediments, supporting the observed geochemical differences. Taxonomic assessment showed the potential impact of anthropogenic pressure along the northern, urbanized shore, as river and river mouth samples revealed high abundances of Bacteroidota, Firmicutes, and Cyanobacteria, which could be attributed to pollution and eutrophication. In contrast, lake sediment communities were dominated by Thermodesulfovibrionia, *Methanomethylicia*, Bathyarchaeia, and Thermoplasmata, suggesting sulfate reducing, thermophilic, acidophilic bacteria and methanogenic archaea to play an important role in tropical lake systems. This study provides novel insights into ecological functions of tropical lakes and contributes to the optimization of management strategies of Lake Sentani, ensuring its holistic preservation in the future.

## Teaser

This study evaluates microbial populations across Lake Sentani, revealing distinct communities in river and lake sediments, suggesting anthropogenic pressure effects, and offers insights for tropical lake management and preservation.

## Introduction

Living microorganisms represent the most significant proportion of biodiversity on Earth and play an essential role in biogeochemical processes across aquatic ecosystems (Tranvik et al. 2009). Microbial communities especially contribute to the nutrient exchange between terrestrial and aquatic ecosystems. They are crucial players in the biogeochemical cycling of organic matter as well as the biodegradation and biotransformation of pollutants (Kirchman 2002, Ruiz-González et al. 2015). Thus, microbial communities are shaped by their surrounding environment and represent major indicators for the state and fitness of a functioning ecosystem (Lau and Lennon 2012, Zaghloul et al. 2020). Understanding the link between ecosystem functioning and distribution of microbial diversity is therefore crucial to predict the response of the ecosystem to a changing environment.

Lakes contribute disproportionately to global elemental cycles and support a wide suite of ecological functions (Tranvik et al. 2009, Anderson et al. 2020). They represent critical freshwater resources and, especially in rural areas, play an important part in providing economic and societal stability.

Climate and anthropogenic impacts are two of the most important factors impacting the health of aquatic ecosystems in general, and lakes specifically (Poff and Day 2002, Paerl et al. 2016). Both factors can directly or indirectly drive the development of richness and diversity of a local microbiome and significantly affect how well an ecosystem functions. Human activities, including land-use changes as well as industrialization, have been shown to seriously threaten the structure and function of aquatic lake ecosystems, resulting in increased nutrient load, algae blooms, and erosion events (Søndergaard and Jeppesen 2007, Qi et al. 2020, Nwosu et al. 2023). Growing populations surrounding lake water systems can trigger a higher anthropogenic nutrient input to the lake, decreasing water quality and diminishing biological diversity (Zan et al. 2012, Gao et al. 2015). The Anthropocene and the global climate crisis pose new challenges to environmental health, particularly in freshwater systems. Evaluating the extent of anthropogenic stressors on lakes, their potential impact on ecosystem health and link to evolutionary changes is therefore pivotal.

About 40% of the lakes on Earth, representing almost one-third of global lake surface area, lie within tropical latitudes and within areas, that have seen rapid changes and increasing levels of urban development in the last decades (Nilssen 1984, Escobar et al. 2020, Marcotullio et al. 2021). It is well understood that tropical ecosystems are different in many ways from those of temperate regions. Warm climate (>18°C, Feeley and Stroud 2018), higher solar radiation, and precipitation rates have essential consequences for biogeochemical processes in tropical ecosystems (Lewis 1987). The lack of seasonal temperature fluctuations leads to a stable temperature gradient in the water column that is not disturbed by changing surface water temperatures. For instance, annual temperatures in Jakarta range from 32.5°C to 37.7°C (Maru and Ahmad 2014). These temperature ranges represent optimum conditions for mesophilic growth over the entire year (Aragno 1981), providing the basis for significant microbial turnover and large biomass production in tropical lake systems.

The water column in a tropical lake is often stratified with a well-mixed oxic surface water (epilimnion) and a denser, sometimes anoxic, deep layer (hypolimnion) (Katsev et al. 2009). There is no turnover in the entire water column because of a lack of major seasonal changes. The stably stratified water column and the anoxic bottom water provide optimal conditions for undisturbed sedimentation due to a lack of resuspension and bioturbating organisms. These unique characteristics of tropical lake systems result in lacustrine surface sediments exhibiting great heterogeneity regarding their geochemical composition, resulting from geographical, hydrological, and topographical variations in the catchment, and are believed to harbor a distinct microbial community susceptible to changes in the physicochemical state of lake sediments (Ramsey et al. 2005, Vuillemin et al. 2017). Their unique characteristics make tropical lakes very sensitive to increases in nutrient supply and susceptible to changes in water quality and biotic communities in response to eutrophication (Molot et al. 2021). Tropical lakes are also more likely to lose deep-water oxygen, as they are permanently stratified. Thus, to maintain a functioning ecosystem, tropical lakes commonly underlie more stringent legal regulation of organic and nutrient loading than temperate lakes (Kilham and Kilham 1990, Lewis 2000, Muvundja et al. 2009, Sepulveda-Jauregui et al. 2018).

Despite the environmental and socio-economic importance of tropical aquatic ecosystems, the role of microbial communities, and the interaction of microbes within complex aquatic networks in tropical regions remain limited, especially when compared to temperate regions (Jørgensen 2000, Humbert et al. 2009, Ruiz-González et al. 2015, Amado and Roland 2017, Meier et al. 2019). Limited work in this research area has focused on biochemical processes at the sediment-water interface (SWI; Steger et al. 2011) and environmental changes (Saarenheimo 2015, Wang et al. 2018, Han et al. 2020). However, only a small number of studies have evaluated microbial composition, diversity, or metabolism in tropical lake ecosystems, emphasizing major differences to temperate regions (Humbert et al. 2009, Tripathi et al. 2012, Rochelle-Newall et al. 2015). Such studies are necessary to assess how microbial communities shape the structure and functioning of topical lake ecosystems, e.g., through primary production, nutrient cycling, or as water quality indicators (Amado et al. 2013). Data from such investigations also provide insights how microorganisms contribute directly and indirectly to ecosystem services in regions around tropical lakes, which often rely on these water bodies for food production and as a source of water, as well as recreation (Sterner et al. 2020).

While bacterial communities in tropical lakes have been proposed to exhibit general similarities to those in temperate lakes, characterized by the frequent identification of Actinobacteria, Proteobacteria, and to a lesser extent Bacteroidetes and Cyanobacteria (Humbert et al. 2009, Moguel et al. 2021), distinct differences have been observed. For example, variations in the composition of Betaproteobacteria were reported by Humbert et al. (2009).

Microbial communities in tropical lakes are shaped by physicochemical conditions in the water column conditions and the surrounding catchment. For example, in stratified Lake Kivu, which contains high concentrations of naturally occurring methane gas (CH_4_), Crenarchaeota are dominant and believed to play an important role in methane production (Llirós et al. 2010). Analysis of sediments from Lake Towuti (Indonesia) revealed exogenous sources of extracellular DNA related to soils, reworked from the ferruginous catchment (e.g. Actinobacteria, Verrucomicrobia, Acidobacteria), while a very limited number of sequences from primary producers (i.e. Cyanobacteria) and high abundances of Chloroflexi and Planctomycetes suggest substantial degradation of sinking organic matter and planktonic microorganisms (Vuillemin et al. 2017).

Current lack of a solid understanding of the role of microbial communities in biogeochemical processes in tropical lakes hinders efforts to manage and conserve lake ecosystems. As lakes located across temperate latitudes have been significantly affected by increases in population density and changes in land use in the catchment (Lewis 2000), the role and impact of such human activities need to be the focal point of ongoing and future research efforts targeting tropical lake systems. To fill the gap in knowledge about microbial communities in tropical, lacustrine sediments and evaluate the effect of urban development and land use around tropical lakes, the microbial community composition and diversity in such ecosystems need to be studied in greater detail.

**Figure 1.**
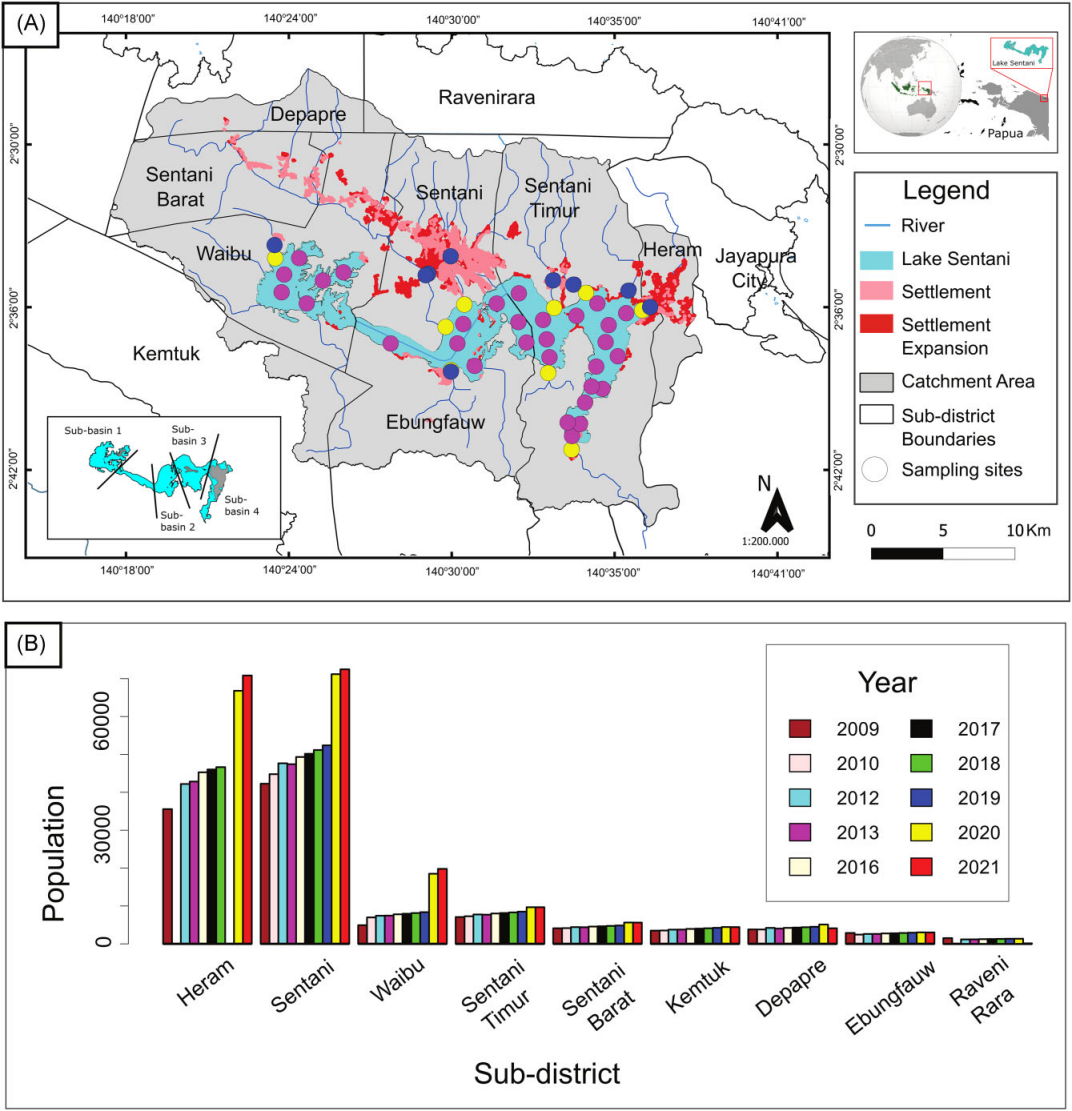
Lake location and setting. (A) Sampling sites and sub-districts in Lake Sentani. Circles indicate sampling sites. Blue, yellow, and purple indicate river, river mouth, and lake sampling sites, respectively. The pink areas indicate settlements prior to 2002, and red areas indicate the settlement expansion from 2002 to 2017. (B) Population by year in sub-districts surrounding Lake Sentani (BPS Kabupaten Jayapura, 2010–2022).

Lake Sentani and its surrounding catchment, located in Papua Province, Indonesia, represent a good candidate for such an assessment. Lake Sentani is characterized by its unique shape, as it is divided into four sub-basins, of which three are separated by shallow sills and one by a narrow natural canal (Sadi 2014, Indrayani et al. 2015a). The catchment is geologically diverse, ranging from carbonates over clastic sediments to igneous and metamorphic rocks (Suwarna and Noya 1995). While the southern shores of the lake are largely rural and only sparsely inhabited, the inhabited areas around Sentani City and Jayapura have constantly expanded. Over the last decade, annual population growth in Jayapura Regency is 2.5% (BPS Kabupaten Jayapura 2020), especially in the sub-districts located in the northern part of the lake (Fig. 1), bringing along an increase in anthropogenic pressure and leading to increased nutrient fluxes to the lake (Walukow et al. 2008). Consequently, indicators for eutrophication, such as cyanobacteria, have already been observed in Lake Sentani over the last years (Pattiselanno 2013, Indrayani et al. 2015b). In addition, land-use changes in Lake Sentani’s catchment have been suggested to cause erosion and flooding (Bungkang et al. 2014, Koyari and Asmaranto 2018, Mujiati et al. 2021).

Some lakes in Indonesia are already facing a massive decline in water quality, indicated by frequent incidents of mass fish deaths. For instance, in Lake Maninjau, West Sumatra, mass fish kills occur frequently. There are numerous fish farms in the lake, thus adding great amounts of nutrients via feeds and fish feces, leading to a deterioration of lake water quality and a buildup of hydrogen sulfide and methane in the sediment. Eventually, sudden outbursts of methane release vast quantities of hydrogen sulfide that cause mass fish kills (Hafrijal 2016, Makmur et al. 2020, Yuniarti et al. 2021). Due to the increasing population around Lake Sentani, there are attempts to increase the fish farming activities in the lake. To anticipate further ecosystem decline, the government of the Republic of Indonesia has selected Lake Sentani as one of the fifteen national priority lakes for being managed and restored (Perpres No. 60 2021).

While recent studies have characterized Lake Sentani’s water column and sediments from a limnological and geochemical perspective (Nomosatryo et al. 2021, 2022), there are currently no data available on microbial diversity in the lake. As microbes play an important role in biogeochemical processes, are highly abundant in aquatic systems, form the bottom of the food web, and are usually very susceptible to environmental changes, they are useful indicators to assess ecosystem health and function (Paerl et al. 2003, Zhang et al. 2023).

We hypothesize that increases in anthropogenic activity over the last decade, particularly along the more populated areas of the catchment, directly or indirectly influence microbial communities in Lake Sentani’s habitats and sub-basins. Consequently, microbial communities residing in areas closer to human development (e.g. draining rivers and shore adjacent) may be more impacted by these events and potentially enriched in coliforms, pathogens, and/or indicators for eutrophication, such as Cyanobacteria. Finally, geological and environmental factors, including water depth, proximity to and runoff from populated areas vs. unpopulated areas are also believed to be driving factors in shaping microbial composition. Although at shallower water depths, the SWI is oxic, most of the sediment is anoxic due to oxygen only penetrating the upper few mm of sediment (Corzo et al. 2018). Therefore, we expect the sediment to be enriched in anaerobic microorganisms.

To address our hypothesis, we employed genomic, 16S rRNA-based analyses to investigate microbial community structure and diversity in surface sediments across Lake Sentani and from ten river and river mouth locations along the shores of Lake Sentani. We conducted an indicator species analysis to detect specific microbial taxa associated with certain habitats or areas of the catchment. We also studied distribution patterns of the microbial populations to understand the niche differentiation potential for microorganisms across the different habitats of Lake Sentani. Using bioinformatic and statistical tools, we were able to evaluate the environmental and anthropogenic impact on Lake Sentani microbial communities.

## Site description

Lake Sentani (2.6°S, 140.5°E) is located near Jayapura, the capital city of Papua Province, and lies at an elevation of 73 m above sea level. The lake covers an area of about 600 km^2^ and is bounded by the Cyclops Mountains to the north (Tappin 2007) and lowlands to the south. The north side of the lake is dominated by volcanic breccia, mafic and ultramafic rocks, and alluvial deposits. In contrast, the southern part of the lake is dominated by limestone of the Jayapura formation, siltstone and claystone of the Makat formation, and alluvial deposits (Fig. SI1.A) (Suwarna and Noya 1995).

There are eight administrative sub-districts located in the catchment area of Lake Sentani (Fig. 1A). Most of the sub-districts located in the northern part of the lake have a higher population than the southern part of the lake (Fig. 1B). Heram and Sentani sub-districts are central settlements in the Lake Sentani catchment area. Furthermore, the Sentani city sub-district along the northern shore (Fig. S1A) is also one of the centers of sago production in the area (the sago palm landscape covers 78 km^2^ around the Lake Sentani watershed), with several thousand tons total of starch production every year (Dimara et al. 2021), as sago is considered one of the local food assets (Sidiq et al. 2021). Sago processing facilities (mills) are located on the northern shore of Lake Sentani, near Sentani City.

At least sixteen rivers drain into the lake (Kementrian Lingkungan Hidup Republik Indonesia 2011, Handoko et al. 2014). The most significant sub-catchment area is the Yahim River, located on the north side of the lake. It covers five sub-districts (Depapre, Sentani Barat also referred to as West Sentani, Waibu, Sentani, and a half of Ravenirara sub-district) and almost 38% of the total catchment area of Lake Sentani (Fauzi et al. 2014). Twelve rivers come from the Cyclops Mountains on the northern side of the lake, and four rivers originate from the lowlands in the south. The Doyo River in the Yahim sub-catchment is the biggest single water source to the lake, with an average discharge of 18.98 m^3^ s^−1^ (Handoko et al. 2014). The only outflow of the lake is the Jayafuri River on the south-eastern tip of sub-basin 4.

The lake is irregularly shaped with approximate dimensions of 28 km (East to West) by 19 km (North to South) and a surface area of 96.3 km^2^ (Kementrian Lingkungan Hidup Republik Indonesia 2011). Lake Sentani consists of four separate basins that are characterized by maximum water depths ranging between 12 and 43 m at the center (own measurements). Literature values range from 30 (Sadi 2014) to 70 m (Indrayani et al. 2015a). Shallow sills with a maximum depth of 6 m connect three sub-basins, the fourth sub-basin is connected via the Simboro Passage, a shallow natural canal (Fig. SI 1).

The geochemical composition of surface sediment and geochemical characteristics of water column and porewater sediment in the four different basins were described by Nomosatryo et al. (2021, 2022). There are differences in the vertical structure of the water column between the sub-basins. The oxycline depth ranges between 25 and 30 m.

## Material and methods

### Sample selection and collection

Surface sediment samples were collected from the upper 3–5 cm from 30 locations within Lake Sentani, using Ekman grabs deployed from a boat. The depth interval was chosen to cover the entire oxygenated part of the sediment (upper few mm) as well as the uppermost anoxic part. Also, previous research showed that geochemical gradients are steepest in this depth interval, indicating high microbial activity (Nomosatryo et al. 2021). Additional to the 30 samples from the lake, nine samples were taken from the river mouth and ten samples from the main rivers that drain into the lake (Fig. 1A), resulting in a total of 49 samples. The samples were transferred into 2 ml screw cap microtubes by using an aseptic spatula. Samples were stored at 4°C during sampling and transportation and frozen at −20°C upon arrival at the InaCC-LIPI (BRIN) laboratory, Cibinong, Indonesia.

### DNA extraction and purification

The total genomic DNA was isolated using the FastDNA TM SPIN Kit for soil (MP Biomedicals, Eschwege, Germany) following the manufacturers’ instructions. Briefly, 0.5 g of sediment and 978 µl of sodium phosphate buffer were added to a screw-cap tube, and homogenization was performed by vortexing at full speed for 10 min. The tubes were then centrifuged at 14 000 *g* for 5 min, and the supernatant was collected. Impurities were precipitated by adding PPS solution. The cleaned DNA lysate was mixed with 1 ml of binding solution, and the resulting mixture was passed through a silica column. Bound DNA was washed with a washing solution containing 70% ethanol and eluted in 80 µl of elution buffer. DNA was extracted from each sample in duplicates, and two extraction blanks were included for each batch of extractions. The quality of the extracted genomic DNA was assessed visually via gel electrophoresis, and DNA concentrations were quantified with the Qubit2 system (Invitrogen, HS-Quant DNA, Waltham, USA).

### DNA amplification and Illumina sequencing

16S rRNA sequence libraries were prepared in duplicate for 49 collected surface sediment samples to assess microbial distribution, diversity, and community composition across the different habitats in Lake Sentani (Table S1, Fig. S2). Unique combinations of tagged 515F (5′-GTGTGYCAGCMGCCGCGGTAA-3′) and 806R (5′-CCGGACTACNVGGGTWTCTAAT-3′) primers targeting the V4 region of the 16S rRNA gene were assigned to each sample, and the targeted region was amplified using polymerase chain reactions (PCR) on a T100^™^ Thermal Cycler (Bio-Rad Laboratories Inc., CA, USA) in 25 µl reactions. Each reaction contained 0.125 µl OptiTaq DNA Polymerase and 2.5 µl 10× Pol Buffer C (Roboklon GmbH, Germany), 1 µl MgCl_2_ (25 mM), 1 µl dNTP Mix (5 mM), 16.625 µl PCR water, each 0.625 µl of forward and reverse primer (20 µM), and 2.5 µl genomic DNA. DNA was diluted 100x prior to PCR to minimize inhibition. The following cycler program was used: an initial denaturing step for 5 min at 95°C followed by 35 cycles of 30 s at 95°C, 30 s at 50°C, and 1 min at 72°C, followed by a final extension step for 7 min at 72°C. PCR reactions were performed in duplicates for all sample extracts and extraction controls. Negative template controls were included for each PCR run. Resulting barcoded PCR products were purified with Agencourt AMPure XP—PCR purification beads using a 2:1 bead-to-sample ratio (Beckman Coulter, Inc., CA, USA). Bead purification was performed for all samples, including controls. DNA concentrations in each cleaned sample were assessed using Qubit Technology. Samples were then normalized according to their concentration and pooled into a single sequencing library to a final concentration of 30 nM. The final 16S rRNA library was sequenced on an Illumina MiSeq platform using 2×300 bp chemistry by Eurofins Genomics Europe Sequencing GmbH (Konstanz, Germany).

### Bioinformatic analysis

The sequencing library was demultiplexed using cutadapt v2.8 (Martin 2011) using the following parameters: -e 0.2 -q 15,15 -m 150—discard-untrimmed, identifying only read pairs with correct barcodes at both ends. The Amplicon sequence variants (ASVs) were generated using the trimmed reads and the DADA2 package v1.18 (Callahan et al. 2016) with R v4.0 using the pooled approach with the following parameters: truncLen=c(240 200), maxN=0, rm.phix=TRUE, minLen=200. Taxonomic assignment was done using DADA2 and the SILVA database v138 (Quast et al. 2013). Sequencing data were uploaded to the European Nucleotide Archive under the accession number PRJEB43748.

### Data processing and in analysis

Absolute singletons, doubletons, and all ASVs with a read count below 20 were removed from the ASV table. Furthermore, ASVs, which were not classified as bacteria or classified as chloroplasts or mitochondria, were also removed from the ASV table. Finally, the ASV table was manually curated for potential contamination using the negative and positive control sequencing results. The ASV table was subsampled to a sequencing depth of 12 000 sequences for alpha diversity analysis. Species richness (S), Shannon index (H), Simpson, and Pielou’s evenness index (J) were calculated using the Vegan Package in R (Oksanen 2015). To evaluate variations across the different sample communities (beta diversity), the ASV table was Hellinger transformed, and a multivariate analysis was conducted. Principal coordinate analysis (PCoA) based on Bray–Curtis distance was used to visualize the dissimilarity of microbial communities between habitats (river, river mouth, and lake). In addition, geochemical measurements (Nomosatryo et al. 2022) were *z*-score transformed and fitted onto the ordination plot of the PCoA as vectors. Differences in microbial community structure between habitats were assessed using analysis of similarity (ANOSIM). The taxonomic relative abundances across samples were visualized through bubble plots using the ggplot2 package. The Venn diagram was created by using the ggVennDiagram package (Gao et al. 2021) to merge ASV readings in each habitat.

### Indicator species analysis

To identify microbial taxa specific to certain samples, an indicator species analysis was conducted. Samples were grouped by their habitat (rivers, river mouths, lakes), their proximity to urban activity (urban, rural), a mixture of both (urban rivers, rural lakes, etc.) and basin (basin 1, 2, 3, or 4, see Fig. 1A). Group assignments of all samples are listed in Table S2. Specialist ASVs were calculated using the “indval” function in the labdsv package (Roberts 2023). Only ASVs with a significant IndVal value (IndVal > 0.8, *P* < .05) and an overall relative abundance of >0.1% across all samples (to avoid very rare taxa) were considered good indicators of specialization for any of the set groups. In contrast, ASVs with high incidence (>80% of the samples) and high relative abundance over all sites (>0.01%) were considered generalists.

## Results

### Data generation and sequencing results

Overall, sequencing generated between 12 729 and 292 628 sequences (post-trimming and contamination removal) per sample, with an average sequencing depth of 132 505 sequences (Table S1). Sequencing resulted in the identification of 56 196 different ASVs across all samples, of which 89.8% were classified as Bacteria and 10.2% were classified as Archaea.

### Sample specific microbial diversity

Assessment of overall microbial diversity revealed an average of 1854 different detected ASVs per 10 000 sequences and an average evenness score of 0.98 (Table S1) per sample. Our analysis suggested microbial diversity parameters (richness and evenness) to be relatively similar across the evaluated habitats (Fig. S3); however, minor variations were observed. Microbial communities across the different river sediment samples were found to be least diverse (Fig. S3, Table S1), and differences in the number of ASVs between river samples and the remaining samples were detected using analysis of variance (ANOVA) (Fig. S3, *P* = .03). Richness of river communities ranged between 793 and 1842 ASVs per 10 000 sequences, with a Shannon Index between 6.50 and 7.38 and an evenness range of 0.96–0.98, respectively. Microbial diversity measurements were moderately higher in river mouth samples than in river samples, ranging between 1246 and 2833 ASVs per 10 000 sequences, resulting in a Shannon Index of 6.95–7.85. Microbial communities in the lake sediments were characterized by the largest range in diversity, with 447–2141 ASVs reported per 10 000 sequences and a Shannon Index of 5.89–7.52. Assessment of species evenness suggested microbial communities in rivers (0.96–0.98), river mouths (0.98–0.99), and lake sediments (0.97–0.98) to be very similar with respect to the distribution of microbial taxa. Comparison of Shannon and Eveness values using ANOVA did not reveal any statistically significant differences (Fig. S3, *P* > .05). Detailed diversity measurements for all habitats, locations, and samples are summarized in the supplemental materials (Table S1).

### Differences between habitat

**Figure 2.**
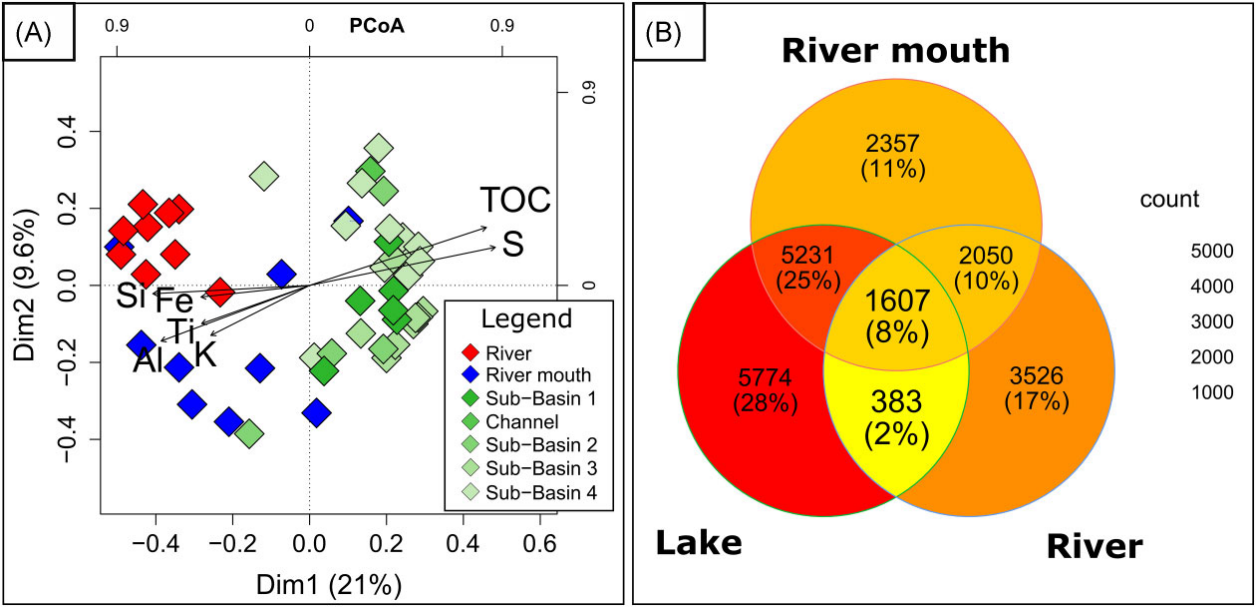
PCoA based on Bray–Curtis distances of microbial community structure (A) and Venn diagram (B). Different colors of the square symbol in PCoA indicate different habitats and solid arrows represent the environmental parameters as variables of geochemical characteristics. Venn diagram expressing the unique and potentially shared ASVs between the river, river mouth, and lake (the total ASV’s of sub-basin 1, channels, 2, 3, and 4).

Ordination analysis using Bray–Curtis dissimilarity indices visualized on a PCoA plot suggested microbial community composition to differ across the evaluated habitat types, as three distinct clusters representing the three sampling locations (river, river mouth, and lake) were observed (Fig. 2A). Visual clustering was supported by statistical calculations using analysis of similarity (ANOSIM), suggesting microbial communities from lake and river sediments to be significantly different (*P* = .001, *R* = 0.98, Table S2). Similarly, lake sediment communities were also found to differ significantly from microbial populations in river mouth samples (*P* = .001, *R* = 0.67, Table S2). Microbial communities in river and river mouth samples varied slightly (*P* = .002, *R* = 0.62, Table S2). Visual inspection did not suggest any differences among microbial composition across the lake basins, as no obvious separation between samples could be observed in the ordination analysis (Fig. 2A). However, ANOSIM calculations emphasized a moderate, but significant dissimilarity in microbial community structure between sub-basin 2 and sub-basin 3 (*P* = .026, *R* = 0.68, Table S2) and small differences between sub-basins 1 and 3 (*P* = .003, *R* = 0.33, Table S2).

Analysis of shared and unique ASVs across the three main Lake Sentani habitats (river, river mouth, lake) and the four different lake sub-basins also emphasized differences in microbial richness among the evaluated sites and highlighted a gradual shift in microbial distribution from river, to river mouth, and lake samples. While 5774 ASVs were exclusively identified in lake sediments, only 1607 ASVs (8%) were found to be shared across all three habitats (Fig. 2B). River and river mouth shared 2050 ASVs, while 5312 ASVs were identified in both river mouth and lake sediments. Only 383 ASVs (3%) occurred in both river and lake sediments.

### Oxygen availability does not significantly affect community structuring

Due to the water depths in the rivers and river mouths being <2 m, we assume oxygen can reach the sediment surface. To assess the effect of oxygen on the microbial community composition, samples were clustered based on the oxic-anoxic conditions (oxygen concentration in bottom waters) in the surface sediment lake and light penetration (euphotic zone, 3 x Secchi depth, 9 m) (Table S2, Fig. S4). Neither visual separation nor statistical evaluation using ANOSIM suggested any significant dissimilarity between oxic and anoxic conditions (Table S2). Nevertheless, we observed a moderate, but insignificant dissimilarity between microbial communities in the euphotic zone (0–9 m depth) and the anoxic zone (*P* = .008, *R* = 0.50, Table S2).

### General community composition

**Figure 3.**
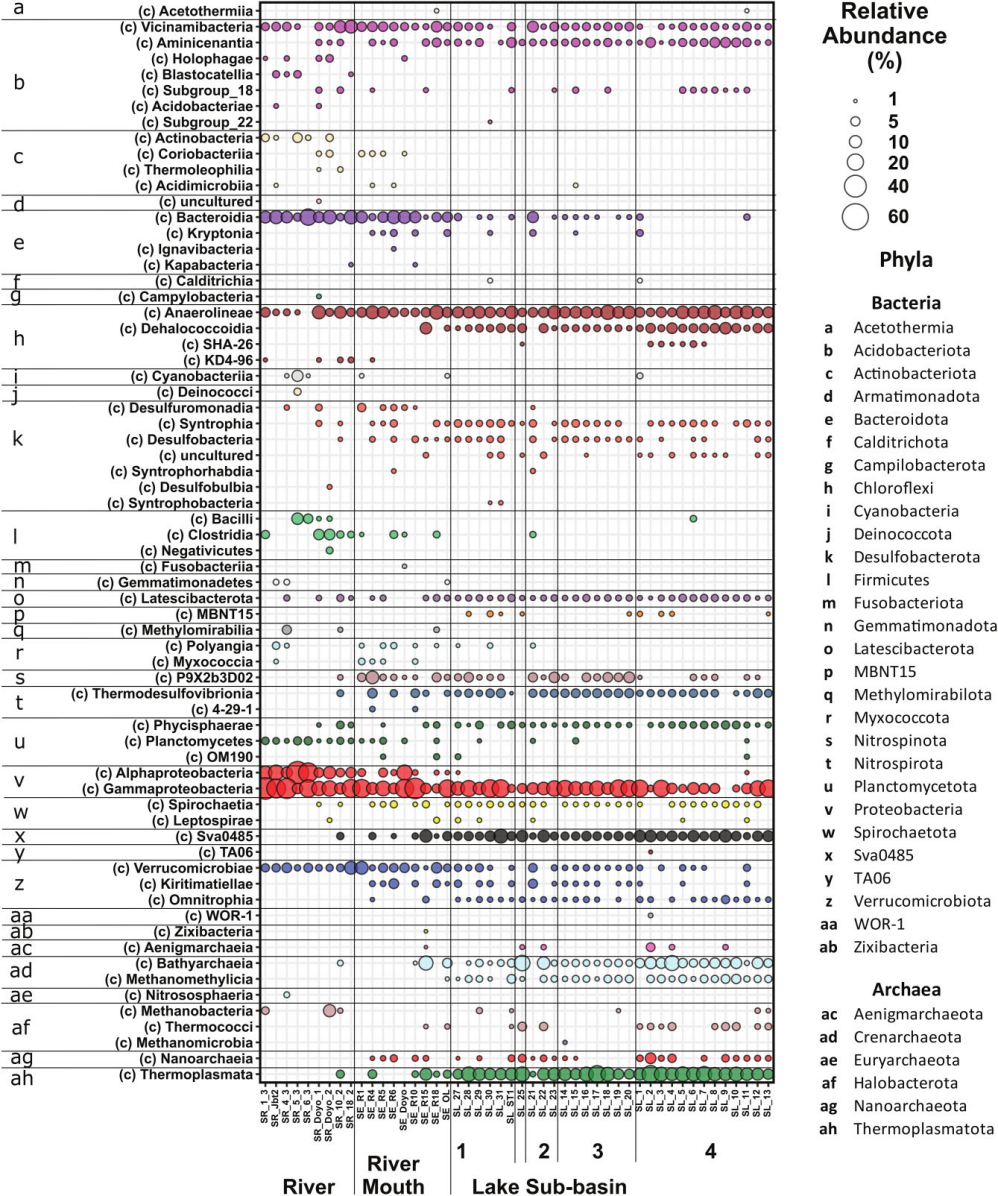
Bubble plot depicting microbial community composition (>1%) in analyzed surface sediment samples at the class level is indicated by bubble colors based on 16S rRNA gene sequencing results. Associated phyla are indicated by lower case letters and listed on the right.

Taxonomic evaluation of the collected sediment samples identified 23 phyla (18 phyla of bacteria and 5 phyla of Archaea) across Lake Sentani’s lake and catchment surface sediment microbial communities (Fig. 3). The majority of the samples were dominated by bacteria (53.3%–99.7%), however, lake sediment samples were characterized by high Archaea abundances (up to 46.7%). Proteobacteria (up to 54.1%), Chloroflexi (up to 19.1%), and Acidobacteria (up to 16.6%) were the most abundant bacterial phyla and were detected in all three habitats (Table S3). Bacteroidota (up to 20.2%) and Alphaproteobacteria (up to 41.6%) were especially abundant in the river and river mouth habitats, whereas sulfate-reducing taxa Desulfobacterota (up to 9.5%) and Thermodesulfovibrionia (up to 4.5%) were particularly enriched in lake sediment samples. Indicator species analysis resulted in the identification of three ASVs that were identified in more than 80% of the evaluated samples with a relative abundance >0.1%. These included an uncultured Thermoplasmata archaeon ASV and two uncultured Gammaproteobacteria ASV, which were classified as Steroidobacteracae (Table S3). To better understand microbial distribution patterns across the three habitats, the microbial community composition was assessed in more detail, particularly at lower taxonomic levels.

### Microbial community of the river sediments

River sediment samples were taken from eight different rivers, six of which are located across the more densely populated north-eastern shore areas of Sentani City and Jayapura. River sediment samples were especially characterized by high abundances of Alphaproteobacteria (up to 41.6%), Gammaproteobacteria (up to 38.8%), Bacteroidia (up to 20.2%), and Clostridia (up to 7.1%).

Closer examination of the taxonomic profiles suggested several Alphaproteobacteria sub-taxa to be especially enriched in river sediments and largely absent from lake samples (Table S3). Sphingomonadaceae (up to 33.9%), including *Novosphingobium*, Xanthobacteraceae (up to 1.4%), and nitrogen-fixing Hyphomicrobiaceae (up to 1.8%) and Rhizobiaceae (up to 0.6%), were found in all river sediments. Caulobacteraceae (up to 1.0%) were notably present in Sentani City sediments (Doyo and SR6, Fig. S1B), while Beijerinckiaceae (up to 0.7%) were identified in Sentani City and southern shore rivers draining into sub-basin 2 (Fig. S5).

Gammaproteobacteria were detected across all river sediments. Uncultured Comamonadaceae (up to 12.7%) were found in all river sediments except those on the southern shore, with *Hydrogenophaga* and *Acidovorax* as dominant genera. Xanthomonadaceae (up to 6.5%) and Rhodanobacteraceae (up to 0.6%), including *Lysobacter, Ahniella*, and *Arenimonas*, were exclusive to northern shore rivers draining into basins two and three. Nitrosomonadaceae (up to 3.5%) were present in northeastern shore rivers and the easternmost river draining into basin 1 but were absent from lake samples.

Bacteroidia were specifically enriched in river sediments, with Chitinophagaceae (up to 8.3%) and Saprospiraceae (up to 2.2%) detected in almost all river samples, consisting of ASVs classified as *Terrimonas* and *Lacibacter* (Kämpfer et al. 2011).

Firmicutes taxa were also exclusive to river sediments. Clostridiaceae (up to 5.4%) were enriched in Doyo and southern shore sediments, while Bacillus were found in northern shore rivers flowing through Sentani City into sub-basins 2 and 3, and the southern rivers discharging into these sub-basins.

Additional river-specific taxa included Chloroflexi, which could be more accurately classified as Anaerolinaeae (up to 11.6%), Planctomycetes of the family Gemmataceae (up to 1.4%), and Acidobacteriota of the family Blastocatellaceae (up to 2.2%), all absent from lake sub-basins (Fig. 3, Table S3). River sediments were the only habitat where Cyanobacteria (up to 8.0%), Deinococci (up to 2.7%), methane-oxidizing Methylomirabilia (up to 4.9%), and archaeal Methanobacterium (up to 9.3%) were detected.

ASV-level indicator species analysis identified only three ASVs with a relative abundance >0.1% that were specifically associated with all river sediments. These included one ASV closely related to Blastocatellaceae, another to Clostridia, and an uncultured Gammaproteobacteria ASV. Additionally, five ASVs were identified as specialists for rivers in urban areas on the northern and eastern shores, including a *Sphingomonas* ASV and an uncultured Omnitrophales ASV (Table S5). In contrast, 36 ASVs were found to enrich microbial communities in rural rivers, including ASVs similar to Vicinamibacter, Chitinophagaceae, *Clostridium*, Xanthobacteraceae, *Chthoniobacter*, and Anaerolineaceae (Table S5).

In summary, river sediment communities were characterized by a high abundance of Alpha- and Gammaproteobacteria, with Firmicutes and Bacteroidetes particularly present at selected sites along the northern shore. Location-driven differences were further highlighted by less frequent taxa, such as Cyanobacteria, which were detected in only one sample.

### Anoxic conditions shape lake sediment communities

Lake sediment samples exhibited distinct microbial community profiles, characterized by anaerobic and autotrophic microorganisms, differing from river sediments particularly at lower taxonomic levels. Lake communities featured high abundances of sulfate-reducing Desulfobacterota (up to 9.0%), acidophilic Euryarchaeota (up to 4.9%), and greater archaeal diversity compared to river and river mouth habitats. Indicator species analysis identified 61 ASVs specifically associated with lake sediments, though none were unique to rural or urban lake sediments (Table S5).

Thermodesulfovibrionia (up to 4.5%) and Deltaproteobacterium *Candidatus* Sva0485 (up to 13.9%) were especially enriched and could be identified in almost every single sample across sub-basins 1, 2, and 3 and in the majority of samples from basin 4 (Fig. 3, Table S3). Both groups include known sulfate-reducing microorganism. Indicators Species analysis suggested three Sva0485 ASVs to be lake sediment specialists (Table S3). Sub-basin 4 communities were also characterized by the frequent detection of Phycisphaerae (up to 3.7%), which are usually associated with marine environments (Youssef et al. 2015). Anaerobic Latescibacterota candidatus taxa P9×2b3D02 sequences were detected across all lake sub-basins (up to 7.4%).

In contrast to river sediments, almost no Alphaproteobacteria were detected in lake sediments, and Gammaproteobacteria signatures were less diverse. We specifically identified ASVs associated with thermophilic, potentially autotrophic and hydrogen utilizing Hydrogenophilaeceae and Rhodocyclaceae [especially in sub-basins 3 (up to 3.7% and 4.7%, respectively) and 1 (up to 1.4% and 4.6%, respectively) and methane oxidizing Methylococcaceae (up to 0.5%)]. Sequences closely related to the facultatively autotrophic sulfur and hydrogen oxidizer *Sulfurisoma* (up to 1%) were detected in a limited number of sub-basin 1 samples and almost all sub-basin 3 lake sediments. Even more than the other two habitats, lake sediments were characterized by Chloroflexi, specifically by Anaerolineae (as the case in river and river mouth sediments) and Dehalococcoidia. Seven ASVs classified as Anaerolineae were determined as lake specialists. Additionally, sequences classified as Aminicenantia, a recently proposed Candidatus class frequently observed in various subsurface marine and non-marine low-oxygen environments, were detected in most lake sediment samples across all four basins (up to 6.0%, Table S3). *Thermoanaerobaculum* (up to 0.5%) was notably identified in all sub-basin 2 samples but was found only sporadically across the remaining habitats.

As stated above, microbial communities in lake sediments distinguished themselves from those of the rivers and river mouths specifically by greater archaeal diversity and abundances. Lake sediments were especially enriched in Bathyarchaeia (up to 17.6%) and *Methanomethylicia* (up to 5.1%) (both Crenarcheota) and Thermoplasmata (up to 23.0%) (Fig. 3). ASVs belonging to all three of those taxa were considered lake sediment specialists (Table S5). In addition, Thermococci (up to 4.4%) and Nanoarchaeia (up to 7.3%) were identified in several samples, mostly in sub-basins 2 and 4. Unlike in the river sediments, no *Methanobacterium* signatures were detected.

One sediment sample was collected from the Simporo channel connecting basins 1 and 2. The microbial community composition in this sample was similar to those identified across the other lake samples but lacked some of the methanogenic and sulfate reducing taxa, particularly Thermodesulfovibrionia and *Methanomethyclicia*. In contrast to the remaining parts of the lake, channel sediments lacked Vicinamibacteria or Aminicenantes signatures, but were characterized by high relative abundances of Bathyarchaeia (17.6%) and Nanoaerchaeia (3.4%).

### Hybrid river mouth communities

Evaluation of river mouth (RM) sediments revealed dynamic microbial communities influenced by both river and lake habitats, depending on the location. A small number of microbial taxa were specifically enriched in river mouth sediments, and six different ASVs were identified as indicators for RM communities (Table S5). Notably, the halorespiring, facultative anaerobic *Anaeromyxobacter* (up to 2.1%) and the thermophilic anaerobic taxon *Geothermobacter* (up to 2.1%) were identified. *Deferrisoma* (up to 0.7%), another thermophilic taxon often associated with hydrothermal vents, was detected in the majority of river mouth samples but was absent on the southern shore and in river mouths or sediments of the outflow.

In contrast, methylotrophic Candidatus *Methanomethylicus* (up to 5.1%) and Latescibacteraceae (up to 8.2%), both of which were frequently and abundantly detected across lake sediments, were only detected in river mouth sediments on the southern shore and sediments from the Jayefuri (alternative spelling: Jaifuri) river at the outflow (Figs S1B and S6A). Similarly, archaeal Thermoplasmata and Bathyarchaeia were abundant in river mouth samples from the same locations. Thermoplasmata were also detected in the sediments of one northern shore river mouth sediments (River 4 draining into sub-basin 4).

The ubiquitous detection of several Bacteroidia taxa suggests that the majority of river mouth sediments are impacted by rivers. We identified Chitinophagaceae sequences (up to 4.1%) in four and Saprospiraceae sequences (up to 1.7%) in six of the nine examined river mouth communities. Four Bacteroidia ASVs were classified as RM specialists, including one Bacteroidetes ASV, one Saprospiraceae ASV, and one Prolixibacteraceae ASV (Table S3). Furthermore, Alpha- and Gammaproteobacteria identified in river sediments were also detected in the corresponding river mouth samples. Notably, elevated levels of Sphingomonadaceae (up to 8.3%), Comamonadaceae (up to 4.2%), and Methylomonadaceae (up to 2.5%) were found in northern shore river mouth sediments. In contrast, Hydrogenophilaceae (up to 1.6%) and Rhodocyclaceae (up to 8.9%), which were more prominent in lake samples, were only detected in a small number of river mouths and the Jayefuri outflow sediment communities.

*Anaerolinea* (class Chloroflexi) were detected in all evaluated river mouth samples, independent of location or habitat (up to 13.0%), while Dehalococcoidia (up to 8.6%) were exclusively detected in river mouth sediments from the southern shore of sub-basin 3 (R15). Finally, sequences affiliated with the candidatus *Omnitrophus* taxon (up to 2.1%), an indicator for anoxic environments with a range of potential metabolic capabilities, including carbon fixation and dissimilatory nitrate reduction (Williams et al. 2021), were identified in all river mouth samples, except one location near Sentani City (Doyo).

These findings suggest that river mouth communities are either driven by the rivers, as shown by the enrichment in Alpha- and Gammaproteobacteria or Bacteroidetes, or, in some locations, driven by lake communities and harbor thermophilic and methanogenic Archaea and sulfate reducers.

## Discussion

Investigations of microbial communities in tropical lake systems and their response to geochemical and anthropogenic processes remain relatively sparse, limiting the current understanding on how ecosystem function, microbial distribution, and diversity are linked in such environments. This is especially concerning as urban development and increases in anthropogenic activity in these historically rural regions are increasing. Lake Sentani and its surrounding areas have experienced rapid population growth over the past decade. These developments, together with the sago production in this area, make this location highly interesting and relevant for such a study. While previous work provided a thorough evaluation of sediments and water columns from a geochemical standpoint (Nomosatryo et al. 2021, 2022), detailed assessment of microbial communities across the diverse Lake Sentani habitats allowed us to examine microbial distribution patterns across a unique tropical lake system and explore if and how far anthropogenic and geogenic features shape this ecosystem.

### Geographical features and general findings

Lake Sentani’s catchment area exhibits varying levels of urban development, with a stark contrast between the northern and southern shores. The area around Sentani City (Sentani sub-district) is the most densely populated, bordering sub-basins 2 and 3. The northern shore, directly adjacent to human settlements and the city limits of Sentani City, experiences more urban influence. Several rivers descending from the Cycloop Mountains run through urban settlements, likely impacting microbial communities in river mouths and nearby lake areas.

In contrast, the western part of Lake Sentani (sub-basin 1) is surrounded by secondary forest, less affected by rivers originating in the Cycloop Mountains, and is relatively isolated, connected to the rest of the lake only through the small, shallow Simboro canal. This area is less populated, though human settlements do exist along the shoreline. The easternmost sub-basin 4 is more exposed to anthropogenic activity in the north, as the western limits of Jayapura City extend to the lake shore, while the southern part remains more rural, with secondary forested shores and only small human settlements.

General findings of this study highlight the large degree of variation in the freshwater bacterial community in taxonomic composition across the different Lake Sentani habitats, as microbial communities in river sediments were especially enriched in Alphaproteobacteria and Bacteroidetes. Lake sediment communities were statistically different and especially enriched in Archaea and sulfate reducers. Indicator Species analysis revealed several ASVs specifically associated with distinct habitats, suggesting especially the lake communities to be characterized by the highest number of such specialized taxa. In addition, more subtle differences in microbial community structure across the sub-basins also suggest that the different environmental and geochemical conditions at each location likely influence the microbial diversity patterns. Several microbial groups, including Gammaproteobacteria and Chloroflexi, could be identified across all habitats, showcasing the interconnectivity of the lake system. Our findings also suggest the river mouth habitats to be a transition zone between the river and lake ecosystems, as the microbial community in this area features a mixture of microorganisms found in the other two habitats, while only a small number of exclusive ASVs could be identified. These observations are consistent with the geochemical characteristics of surface sediments in this habitat as reported previously (Nomosatryo et al. 2022).

### Anthropogenic activities may drive northern shore river and river mouth communities

**Figure 4.**
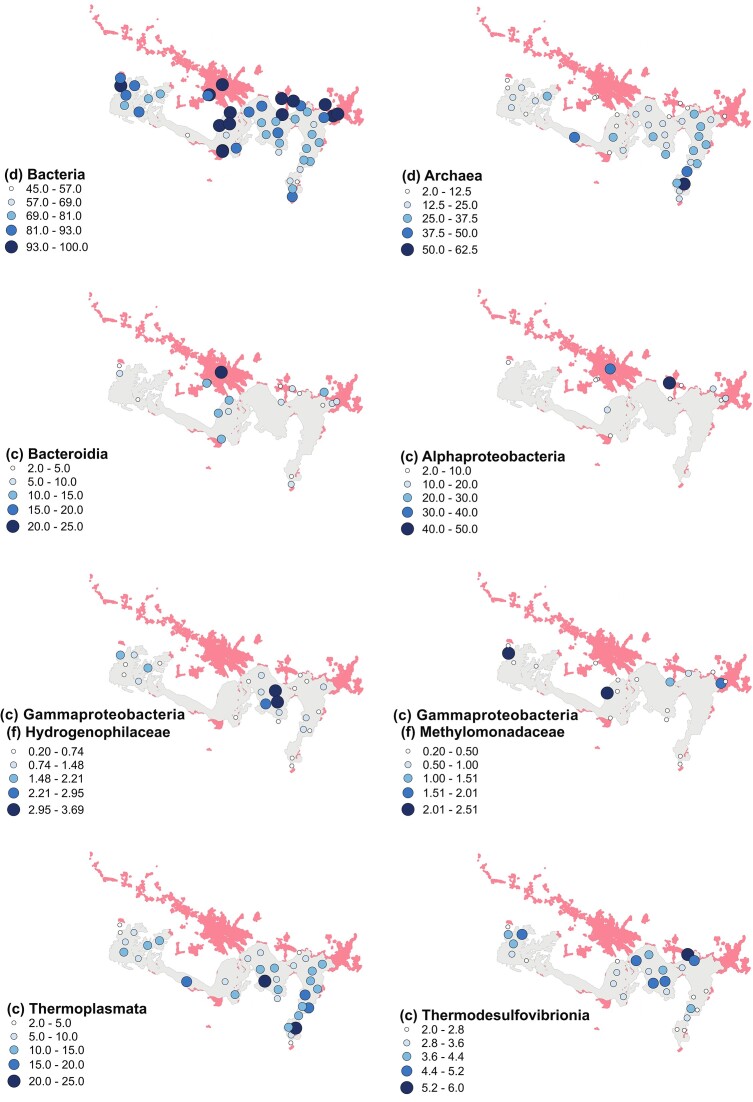
Spatial distribution of selected relevant and abundant microbial taxa. The distribution is shown as relative abundance in %. The red color indicates the settlement area, and the gray color represents the water body of the lake.

One of the central objectives of this study was to evaluate distribution and composition of microbial communities across Lake Sentani with a special focus on the role anthropogenic pressures might play. We hypothesized that particularly river and lake sediments found along the more populated northern shore are characterized by microbial populations often associated with anthropogenic pressure. Our results partially confirmed this hypothesis, as especially sediment samples taken from rivers and river mouths located along the northern shore and thus running through the populated areas of Sentani City and areas close to Jayapura City were enriched in certain microbial groups, including Bacteroides and Firmicutes (Fig. 4). Although our indicator species analysis identified a small number of ASVs that were exclusively associated with more urban river or lake samples, none of them stood out as clearly pathogenic or seemed to be closely linked to anthropogenic activity.

While the population density and rate in the Lake Sentani area are still relatively low compared to average values across other areas in Indonesia (Kementerian Lingkungan Hidup Republik Indonesia 2011), the local populations in those sub-districts located within the Lake Sentani catchment area are increasing (Fig. 1B), especially in the sub-districts Sentani and Heram, and thus might directly or indirectly influence the river and lake water conditions. Closer taxonomic evaluation suggested the majority of identified Bacteroidetes signatures to belong to the genera *Terrimonas* and *Lacibacter* and thus represent common soil and sediment bacteria, while *Bacillus* and *Clostridium* were the most abundant Firmicutes in these river sediments. However, no Firmicutes or Bacteroidia ASVs did statistically stand out as an indicator for “urban” samples. Nevertheless, their increased abundance in river and lake samples around more populated could still serve as an indicator for anthropogenic input. While these organisms do not serve as classic fecal coliforms, such as *Escherichia coli* or Enterococci, as one may have expected, some can be considered indicators for human and potentially industrial activity, as they are often identified in mammals (Shanks et al. 2011) and have been observed in other polluted lake and surface water environments (Ouyang et al. 2020, Zhang et al. 2020, Gao et al. 2022). Thus, the frequent identification of Bacteroidota and Firmicutes ASVs in river, but also river mouth habitats that lie in close proximity to the urban region of Lake Sentani might be caused by the untreated wastewater from residential areas containing pollutants like nitrates, ammonia, and feces.

Anthropogenic pressure on an ecosystem can also cause elevated nutrient loads resulting in increased carbon and nitrogen turnover, causing eutrophication. Eutrophication in Lake Sentani was reported recently (Pattiselanno 2013, Indrayani et al. 2015a), suggesting the region’s anthropogenic activity might lead to nutrient loading and domestic waste pollution in the river-lake system (Pattiselanno 2013).

Our study supports that especially northern shore river and river mouth sediments may be hotspots of microbially mediated carbon (C) and nutrient processing, as *Novosphingobium, Dechloromonas*, and *Hydrogenophaga*, which are often associated with industrial and/or synthetic wastewaters (Bruce et al. 1999, Yoon et al. 2008), were detected frequently. While there is no major industry that could be considered as a point source for industrial wastewater in the catchment area of Lake Sentani (BPS Kabupaten Jayapura, 2022). There are smaller scattered operations [e.g. Sago flour mill (Dimara et al. 2021)] as well as the major airport runway sitting along the northern shoreline. In an alternative scenario, the increased levels of Alphaproteobacteria might simply be caused by elevated nutrient availability, as reported in the Mulargia River, Italy (Zoppini et al. 2010) and urban lakes in China (Zhao et al. 2016, Cao et al. 2018). Organic matter concentrations in the main river and river mouth sediments along the northern shore were found to be relatively low (average around 1.5%_dwt_) compared to the rest of the lake (average around 11.76%) (Nomosatryo et al. 2022) and other tropical lakes e.g. eutrophic lake Maninjau (21%–34%) and Rawa Pening (62.0%–74.8%) (Purnomo et al. 2013, Dianto et al. 2020). The low OM in this area could be attributed to the steep and intermittent slopes found in the northern region of Lake Sentani, leading to increased erosion from the Cyclops Mountains and a general higher lithogenic input in this area providing a masking effect. Still, these concentrations are still sufficiently high to provide an abundantly available energy source for heterotrophic bacteria across the river system of Lake Sentani.

Another type of microorganisms that was strongly associated with the northern shore and mostly enriched in sediments from Sentani City and Jayapura River sites was the Planctomycetes. This widely distributed group includes common anammox bacteria, is found in marine and freshwater ecosystems, and has an important role in organic matter decomposition (Lindeman 1942, Tank et al. 2010, Zeglin 2015). The presence of Planctomycetes is a potential indicator for ammonia oxidation, which could be driven by increased ammonia availability from agricultural and municipal runoff. Similarly, exclusive Cyanobacteria and Chloroflexi signatures in sediments from rivers located around Sentani City suggest a link to anthropogenic activity. Cyanobacteria are very common indicators for increased nutrient availability and are often associated with eutrophication and pollution events (Cottingham et al. 2015, Huisman et al. 2018). Finally, sediments from the Doyo River site in Sentani City were found to harbor populations of Holophagae and *Clostridium* sensu stricto. Holophagae are known for their ability to anaerobically degrade aromatic compounds and can produce volatile sulfur compounds, while *Clostridum* sensu stricto are common gut bacteria (Alou et al. 2018). Several ASVs belonging to these groups were also found to be statistical indicators for rivers; however, none could be statistically linked to urban rivers, suggesting that at least on the lowest taxonomic level no clear specialists and thus indicators for potential anthropogenic activity were present. Nevertheless, the more predominant presence of the above-described taxa in samples from the more urbanized locations around Lake Sentani could still be considered early indicators of anthropogenic input.

### Limited evidence for anthropogenic impact in lake sediments

Microbial communities in the lake sediments show a different structure compared to those in the river and river mouth, exhibiting few to none of the taxa associated with anthropogenic stress. The only lake samples to harbor any Firmicutes (such as Clostridia) or Bacteroidetes (Chitinophagaecae) signatures were identified in sub-basins two and four, mostly in samples taken closest to the northern shore, and thus in proximity to the most urbanized areas of Lake Sentani. While overall only a limited number of samples falls in this category, these observations could represent the first traces of anthropogenic impact on the lake. The city of Sentani is adjacent to sub-basins 2 and 3; hence, most of its wastewater discharges into these sub-basins, which are considerably different from each other. Sub-basin 2 is the only sub-basin in Lake Sentani to have a fully oxidized water column (Fig. S1B); hence, organic matter deposited on the lake floor will be degraded aerobically, which is much more efficient than anaerobic degradation (Kristensen et al. 1995). In sub-basin 3, with a much greater water depth and anoxic bottom waters, we measured on average higher TOC concentrations than in sub-basin 2 (Nomosatryo et al. 2022). Additionally, temporal assessments to evaluate further dispersal of these taxa (such as Clostridium and Bacillus, which can be indicators or anoxic waters and thus eutrophication) throughout the lake sediments are thus highly recommended to estimate the successive anthropogenic impact and to assess how eutrophication and physicochemical conditions in the lake are shaping microbial community composition.

Analysis of the remaining lake data suggested the majority of microbial population composition and dynamics to be primarily driven by oxygen concentrations or rather redox potential. The organic matter concentration in Lake Sentani is quite variable (1.5–27.9% dw) (Nomosatryo et al. 2022), its degradation leading to vastly different oxygen demands and hence, in combination with the variable bathymetry of the lakes’ sub-basins to different scenarios with regard to bottom water oxygenation and stability of a monimolimnion, i.e. permanently anoxic bottom waters (Nomosatryo et al. 2022). But even in those cases where the SWI is oxic, oxygen penetration depths in these sediments are usually <5 mm (Corzo et al. 2018).

Therefore, anaerobic processes are prevalent in the sediments across the sampled lake locations. Consequently, the frequent observation of anoxic fermenters such as Crenarchaeota, Thermoplasmatota, or Acidobacteriota in lake sediments was expected. Especially notable was that numerous ASVs belonging to these taxa were strongly linked to the lake habitat and were suggested to be strong indicators for lake sediments, in contrast to river and river mouth sediments. These microbial distribution patterns are similar to those observed in the iron-rich methanic sediments of Lake Kinneret, Israel, where microorganisms fermenting amino acids and other products of necromass degradation are abundant and include taxa like Anaerolineaceae, Thermodesulfovibrionia, SVA0485, and Bathyarchaeia (Elul et al. 2021).

In addition, the dominance of *Anaerolinea* (Chloroflexi), *Thermodesulfovibrionia* (Nitrospirae), and Sva0485 in the lake sediments emphasizes the high potential for dissimilatory sulfate reduction. Similar microbial groups also contribute to sulfate reduction in ferruginous Lake Towuti, which has sulfate concentrations that are usually below 10 µM (Vuillemin et al. 2018). With generally higher sulfate concentrations of up to 40 µM (Nomosatryo et al. 2021), these processes likely play an even bigger role in Lake Sentani.

### Methanogens and thermophiles shape archaeal Lake Sentani communities

Besides sulfate-reducing taxa, lake sediments were especially abundant and diverse in Archaea (Fig. 4). The presence of strict or facultative anaerobes, like methanogenic Methanomethylicia, thermophilic Thermococci, as well as Thermoplasmata and Bathyarchaeia, which have also been suggested to contribute to methane production (Offre et al. 2013, Romano et al. 2021), are indicative of the limited oxygen availability in the sediments. Our results suggest methane production in Lake Sentani sediments to be mostly driven by methylotrophic methanogens, classified as *Candidatus* Methanomethylicus and Thermoplasmata, of which no representatives have been isolated so far, but which are believed to contribute to methane production by potentially re-mineralizing organic matter to methane as their genomes have been proposed to encode distinct methyl-coenzyme M reductase genes (Clesceri et al. 1998, Poulsen et al. 2013, Ji et al. 2016, Moguel et al. 2021). In addition, decaying aquatic plants instead of anthropogenic sources may be the main methylated compound source for the found Methylotrophic methanogens in the present study due to the more anoxic conditions. The occurrence of Thermoplasmata across all sub-basins suggests their adaptation to this tropical lake environment. Thermoplasmata are known for their acidophilic and thermophilic traits, which likely contribute to their prevalence in sediment ecosystems within tropical lakes in general. Furthermore, certain groups have the metabolic capability for methane production, solidifying their role as potential contributors to biogeochemical processes within these environments. Another group of Archaea identified in the majority of analyzed lake samples, and especially abundant in basin 4, were the Bathyarchaeota. This group has previously been detected in high abundance in sediments of tropical lakes (Vuillemin et al. 2018, Zhou et al. 2018, Romano et al. 2021) and is generally widely distributed across nutrient-poor marine and terrestrial sediments. Certain members of this Candidatus taxon are believed to fix CO_2_ via the Wood–Ljungdahl pathway and acetogenesis (He et al. 2016). While the ability of Bathyarchaeota to carry out methanogenesis has not been proven through direct measurements of metabolism, genomic data suggest several members of this group have the ability to form methane from methanol and methylamines (Evans et al. 2015, Zhou et al. 2018). Their ubiquitous presence in Lake Sentani further supports their role in tropical lakes and highlights the need to evaluate their metabolism and role in biogeochemical interactions in more detail. Notable was the detection of extremophilic, organotrophic sulfidogenic *Thermococcus* in certain basin locations, a genus usually found in higher temperature environments. Some *Thermococcus* species can produce O_2_, H_2_, and H_2_S, which may be used by autotrophic neighbors such as methanogens (Cho et al. 2017). Interestingly, *Thermococcus* occurred together with Nanoarchaeia, another closely related thermophile known for its occurrence in very high-temperature environments, but which lacks the ability to utilize sulfur species or H_2_ (Brochier et al. 2005).

Overall, the wide distribution of distinct archaeal taxa across Lake Sentani sediments agrees with observations made for water column and sediment samples in other tropical lakes, such as Lake Towuti and Lake Kivu (İnceoğlu et al. 2015). Our findings suggest an overall, high potential for methane production from these sediments, not just from classical methanogens, while at the same time identifying several chemo- and autotrophic archaeal groups, which could be indicators for a variety of metabolic processes occurring within lake sediments. This means our data underline the role of thermophilic, methanogenic, and acidophilic archaeal groups in natural, undisturbed tropical lake settings and highlight an elevated potential for methane production, but also the generation of CO_2_ and sulfide.

### Geographic features translate to microbial distribution across lake basins

Even though lake sediments across the four major sub-basins share a lot of similar microbial taxa, few but distinct differences could be observed, suggesting that each sub-basin’s geogenic but also anthropogenic features may play an important role. As discussed above, the only lake samples to harbor any Firmicutes or Bacteroidetes signatures were identified in sub-basins 2 and 4, suggesting these lake areas to be impacted by the first on-sets of anthropogenic stress.

The shallow channel connecting sub-basins 1 and 2 especially stands out, as microbial populations are less diverse overall and lack taxa like Thermodesulfovibrionia and *Syntrophia*, both sulfate reducers, or *Methanomethylicus*, a relatively newly described methylotrophic methanogen (Vanwonterghem et al. 2016), which was observed across the other basins. On the contrary, Bathyarchaeia and Thermococci were most abundant in this shallow and organic-rich environment. The channel has a depth of ∼4 m, and its unique features are the high TOC (27.1%) and high relative distribution of reduced sedimentary sulfur compounds (mainly pyrite) of up to 5 wt%, compared to the other locations (Nomosatryo et al. 2022). An accumulation of decaying aquatic plants most probably causes the high TOC concentration in this channel, and even though oxygen could penetrate to the bottom, elevated sediment pore water sulfide concentrations at this location indicate the surface sediment to be anoxic (Nomosatryo et al. 2021). Such conditions should make this a suitable habitat for methanogens. With classical methanogens being absent, Bathyarchaeia might be the microorganisms filling these roles in terms of anaerobic carbon mineralization. In addition, high abundances of chemotrophic Thermococci could be driven by the high TOC levels in this habitat.

While our data suggest northern shore river and river mouth communities to be impacted by anthropogenic activity and urban runoff, samples from other locations in the Lake Sentani catchment area were found to harbor distinct microbial populations that are more representative of those found in the anoxic lake sediments, including sulfate reducers and methanogens. Especially communities in river and river mouth samples draining into the more secluded sub-basin 1 fall into this category. Similarly, southern river mouths and river communities, which drain into sub-basins 2 and 3 seem less diverse, compared to those found on the northern shore. Communities found in river mouth samples lack the abundant Bacteroidetes and Proteobacteria signatures that were observed in the remaining river mouth samples and could be the result of anthropogenic stressors. In contrast, the two southern river mouth populations were enriched in *Candidatus* Methanomethylicus, which was only identified in two of the three southern river mouth communities. The detection of this methylotrophic methanogen suggests these river mouth habitats are likely more influenced by lake communities, where methanogens are more abundant.

## Conclusion

This study provides crucial information for understanding the Lake Sentani ecosystem under changing environmental conditions in the catchment area due to growing environmental and anthropogenic pressure. In conjunction with our previous work on Lake Sentani, data from this study offer valuable insights into a tropical lake system, highlighting the different microbial communities occurring across the river and lake ecosystems and how they may be impacted by surrounding activities. Our results suggest that microbial communities in rivers are directly influenced by anthropogenic pressure and differ from those in lake sediments. Our study also indicated that increased anthropogenic stressors, likely caused by population growth and urbanization, especially along the northern and northeastern parts, are reflected in the microbial community composition. While only a small number of statistically significant indicator ASVs for those sediments could be identified, and none could be clearly linked to pollution from anthropogenic activity, we were able to identify differences in community composition. River and river mouth sediments, particularly from these more urbanized areas, were enriched in microbial taxa frequently associated with eutrophication and affiliated with human and industrial pollution. In contrast, lake sediment microbial communities were found to reflect the anoxic conditions in the sediments.

## Supplementary Material

fiae162_Supplemental_File

## Data Availability

The generated genomic data was despoiled at the European Nucleotide Archive (ENA) under the accession number PRJEB43748.
