# Supplementary material for: The role of anthropogenic influences on a tropical lake ecosystem and its surrounding catchment: a case study of Lake Sentani
Source: FEMS Microbiol Ecol. 2024 Dec 17;101(1):fiae162. doi: 10.1093/femsec/fiae162 (PMC11707878; doi:10.1093/femsec/fiae162)
Supplement: fiae162_Supplemental_File [file fiae162_supplemental_file.docx]

**-The role of anthropogenic influences on a tropical lake ecosystem and its surrounding catchment: A case study of Lake Sentani**

**Supplemental Information:**

Sulung Nomosatryo^1,2^, Daniel Lipus^1,5^, Alexander Bartholomäus^1^, Cynthia Henny^2^, Iwan Ridwansyah^2^, Puguh Sujarta^3^, Sizhong Yang^1^, Dirk Wagner^1,4^, and Jens Kallmeyer^1^

^1^ GFZ German Research Centre for Geosciences, Section Geomicrobiology, Potsdam, Germany

^2^ Research Center for Limnology and Water Resources, National Research and Innovation Agency (BRIN), KST Soekarno, Jalan Jakarta-Bogor KM 46, Cibinong - Bogor, 16911 Indonesia

^3^Cendrawasih University, Department of Biology, Faculty of Mathematics and Natural Sciences, Jl. Kamp.Wolker, Waena, Jayapura 99358, Indonesia.

^4^ University of Potsdam, Institute of Geosciences, Potsdam, Germany

^5^ Department of Biological and Chemical Sciences, College of Life Sciences, Thomas

Jefferson University, Philadelphia, Pennsylvania, USA

**Tables**

**Table SI 1**. Total number of reads (post trimming) and alpha diversity results for all collected and analyzed samples. S= Species Richness, H= Shannon Indices, J= Evenness Indices

| **Habitat** | **Location** | **DNA (ng/µL)** | | **Total reads** | **S** | **H** | **J** |
| --- | --- | --- | --- | --- | --- | --- | --- |
|  |  | **Post Extraction** | **Post PCR** |  |  |  |  |
| River | SR_1_3 | 130 | 3.60 | 116268 | 1341 | 6.967 | 0.9675 |
|  | SR_10_2 | 31.4 | 2.74 | 52152 | 2049 | 7.4957 | 0.983 |
|  | SR_18_2 | 31.9 | 3.68 | 96023 | 1542 | 7.1286 | 0.9711 |
|  | SR_4_3 | 70.6 | 6.31 | 114702 | 1929 | 7.4084 | 0.9793 |
|  | SR_5_3 | 15.1 | 4.50 | 140226 | 1758 | 7.2609 | 0.9718 |
|  | SR_6_3 | 17.8 | 4.29 | 136948 | 1187 | 6.814 | 0.9625 |
|  | SR_Doyo_1 | 72.55 | 3.76 | 39351 | 1460 | 7.1051 | 0.9751 |
|  | SR_Doyo_2 | 22.35 | 4.20 | 101930 | 1841 | 7.3314 | 0.9752 |
|  | SR_Jbt2 | 80.2 | 4.22 | 225572 | 2408 | 7.6457 | 0.9819 |
|  | Min | 15.1 | 2.74 | 39351 | 1187 | 6.814 | 0.9625 |
|  | Max | 130 | 6.31 | 225572 | 2408 | 7.6457 | 0.983 |
|  | Median | 31.9 | 4.20 | 114702 | 1758 | 7.2609 | 0.9751 |
|  | Q1 | - | - | - | 1460 | 7.1051 | 0.9711 |
|  | Q3 | - | - | - | 1929 | 7.4084 | 0.9793 |
|  | Q3-Q1 | - | - | - | 469 | 0.3033 | 0.0082 |
| River mouth | SE_Doyo | 20.05 | 3.80 | 97760 | 2226 | 7.5605 | 0.9809 |
|  | SE_OL | 75.7 | 4.01 | 46793 | 1463 | 7.111 | 0.9757 |
|  | SE_R1 | 63.5 | 7.36 | 169342 | 1907 | 7.3854 | 0.9778 |
|  | SE_R10 | 125 | 4.69 | 115916 | 1723 | 7.2803 | 0.977 |
|  | SE_R15 | 33.8 | 4.45 | 169467 | 2434 | 7.6561 | 0.9819 |
|  | SE_R18 | 50.2 | 2.53 | 235214 | 2065 | 7.4956 | 0.982 |
|  | SE_R4 | 78.8 | 3.64 | 181165 | 2153 | 7.5195 | 0.9798 |
|  | SE_R5 | 43.6 | 5.90 | 250876 | 3068 | 7.932 | 0.9879 |
|  | SE_R6 | 62.1 | 3.60 | 102093 | 1965 | 7.4182 | 0.9782 |
|  | Min | 20.05 | 2.53 | 46793 | 1463 | 7.111 | 0.9757 |
|  | Max | 125 | 7.36 | 250876 | 3068 | 7.932 | 0.9879 |
|  | Median | 62.1 | 4.01 | 169342 | 2065 | 7.4956 | 0.9798 |
|  | Q1 | - | - | - | 1907 | 7.3854 | 0.9778 |
|  | Q3 | - | - | - | 2226 | 7.5605 | 0.9819 |
|  | Q3-Q1 | - | - | - | 319 | 0.1751 | 0.0041 |
| Sub-basin 1 | SL_27 | 37.7 | 2.55 | 104852 | 2393 | 7.6478 | 0.983 |
|  | SL_28 | 100.5 | 3.56 | 65893 | 1263 | 6.9352 | 0.9711 |
|  | SL_29 | 15.5 | 5.11 | 165368 | 2501 | 7.6806 | 0.9816 |
|  | SL_30 | 108 | 3.52 | 264922 | 1952 | 7.4102 | 0.978 |
|  | SL_31 | 65.6 | 5.26 | 58015 | 1012 | 6.6887 | 0.9666 |
|  | SL_ST1 | 14.2 | 1.79 | 141358 | 2210 | 7.5601 | 0.9817 |
|  | Min | 14.2 | 1.79 | 58015 | 1012 | 6.6887 | 0.9666 |
|  | Max | 108 | 5.26 | 264922 | 2501 | 7.6806 | 0.983 |
|  | Median | 51.65 | 3.54 | 123105 | 2081 | 7.4852 | 0.9798 |
|  | Q1 | - | - | - | 1435.25 | 7.0539 | 0.9729 |
|  | Q3 | - | - | - | 2347.25 | 7.6259 | 0.9817 |
|  | Q3-Q1 | - | - | - | 912 | 0.5719 | 0.0088 |
| Shallow Channel | SL_25 | 57.6 | 4.58 | 121234 | 1644 | 7.211 | 0.9738 |
|  | Min | 57.6 | 4.58 | 121234 | 1644 | 7.211 | 0.9738 |
|  | Max | 57.6 | 4.58 | 121234 | 1644 | 7.211 | 0.9738 |
|  | Median | 57.6 | 4.58 | 121234 | 1644 | 7.211 | 0.9738 |
|  | Q1 | - | - |  | 1644 | 7.211 | 0.9738 |
|  | Q3 | - | - |  | 1644 | 7.211 | 0.9738 |
|  | Q3-Q1 | - | - |  | 0 | 0 | 0 |
| Sub-basin 2 | SL_21 | 73.7 | 5.17 | 175763 | 2283 | 7.5852 | 0.9809 |
|  | SL_22 | 46 | 5.75 | 88607 | 1547 | 7.1534 | 0.974 |
|  | SL_23 | 52.2 | 2.09 | 130261 | 1818 | 7.3202 | 0.9753 |
|  | Min | 46 | 2.09 | 88607 | 1547 | 7.1534 | 0.974 |
|  | Max | 73.7 | 5.75 | 175763 | 2283 | 7.5852 | 0.9809 |
|  | Median | 52.2 | 5.17 | 130261 | 1818 | 7.3202 | 0.9753 |
|  | Q1 | - | - | - | 1682.5 | 7.2368 | 0.9747 |
|  | Q3 | - | - | - | 2050.5 | 7.4527 | 0.9781 |
|  | Q3-Q1 | - | - | - | 368 | 0.2159 | 0.0034 |
| Sub-basin 3 | SL_14 | 50.6 | 6.31 | 149733 | 2062 | 7.4638 | 0.978 |
|  | SL_15 | 38.1 | 4.39 | 84068 | 1875 | 7.3825 | 0.9796 |
|  | SL_16 | 32.1 | 8.26 | 144947 | 1868 | 7.3604 | 0.9771 |
|  | SL_17 | 53.9 | 4.79 | 165352 | 1924 | 7.3766 | 0.9755 |
|  | SL_18 | 63.2 | 2.59 | 125043 | 1787 | 7.3139 | 0.9767 |
|  | SL_19 | 50.7 | 3.85 | 165829 | 1744 | 7.2755 | 0.9748 |
|  | SL_20 | 104 | 3.76 | 184765 | 1877 | 7.352 | 0.9754 |
|  | Min | 32.1 | 2.59 | 84068 | 1744 | 7.2755 | 0.9748 |
|  | Max | 104 | 8.26 | 184765 | 2062 | 7.4638 | 0.9796 |
|  | Median | 50.7 | 4.39 | 149733 | 1875 | 7.3604 | 0.9767 |
|  | Q1 | - | - | - | 1827.5 | 7.3329 | 0.9754 |
|  | Q3 | - | - | - | 1900.5 | 7.3795 | 0.9776 |
|  | Q3-Q1 | - | - | - | 73 | 0.0466 | 0.0022 |
| Sub-basin 4 | SL_1 | 11.5 | 1.24 | 180915 | 1918 | 7.3837 | 0.9768 |
|  | SL_2 | 39.4 | 8.40 | 12729 | 536 | 6.0646 | 0.9651 |
|  | SL_3 | 67.2 | 7.38 | 203279 | 1745 | 7.2699 | 0.9739 |
|  | SL_4 | 43.4 | 1.33 | 104915 | 1413 | 7.0707 | 0.9748 |
|  | SL_5 | 87.5 | 3.52 | 128142 | 2145 | 7.5084 | 0.9788 |
|  | SL_6 | 49.2 | 5.22 | 108849 | 1774 | 7.3046 | 0.9764 |
|  | SL_7 | 62.8 | 1.99 | 126648 | 1865 | 7.3577 | 0.977 |
|  | SL_8 | 28.7 | 3.97 | 68304 | 1583 | 7.1923 | 0.9763 |
|  | SL_9 | 24.2 | 4.37 | 91044 | 2022 | 7.4635 | 0.9805 |
|  | SL_10 | 36.9 | 1.83 | 39718 | 1491 | 7.1308 | 0.9759 |
|  | SL_11 | 57.15 | 3.46 | 86877 | 2154 | 7.5382 | 0.9822 |
|  | SL_12 | 36 | 6.10 | 188415 | 1972 | 7.4061 | 0.9762 |
|  | SL_13 | 41.15 | 4.52 | 292628 | 2103 | 7.4742 | 0.9769 |
|  | Min | 11.5 | 1.24 | 12729 | 536 | 6.0646 | 0.9651 |
|  | Max | 87.5 | 8.40 | 292628 | 2154 | 7.5382 | 0.9822 |
|  | Median | 41.15 | 3.97 | 108849 | 1865 | 7.3577 | 0.9764 |
|  | Q1 | - | - | - | 1583 | 7.1923 | 0.9759 |
|  | Q3 | - | - | - | 2022 | 7.4635 | 0.977 |
|  | Q3-Q1 | - | - | - | 439 | 0.2712 | 0.0011 |

**Table SI 2.** Relative abundance (%) of microbial community in Lake Sentani at Phyla Level.

**Table SI 3A.** Relative abundance (%) of microbial community in Lake Sentani at Class Level (Bacteria).

**Table SI 3B.** Relative abundance (%) of microbial community in Lake Sentani at Class Level (Archaea).

**Figures:**

**A**

**B**


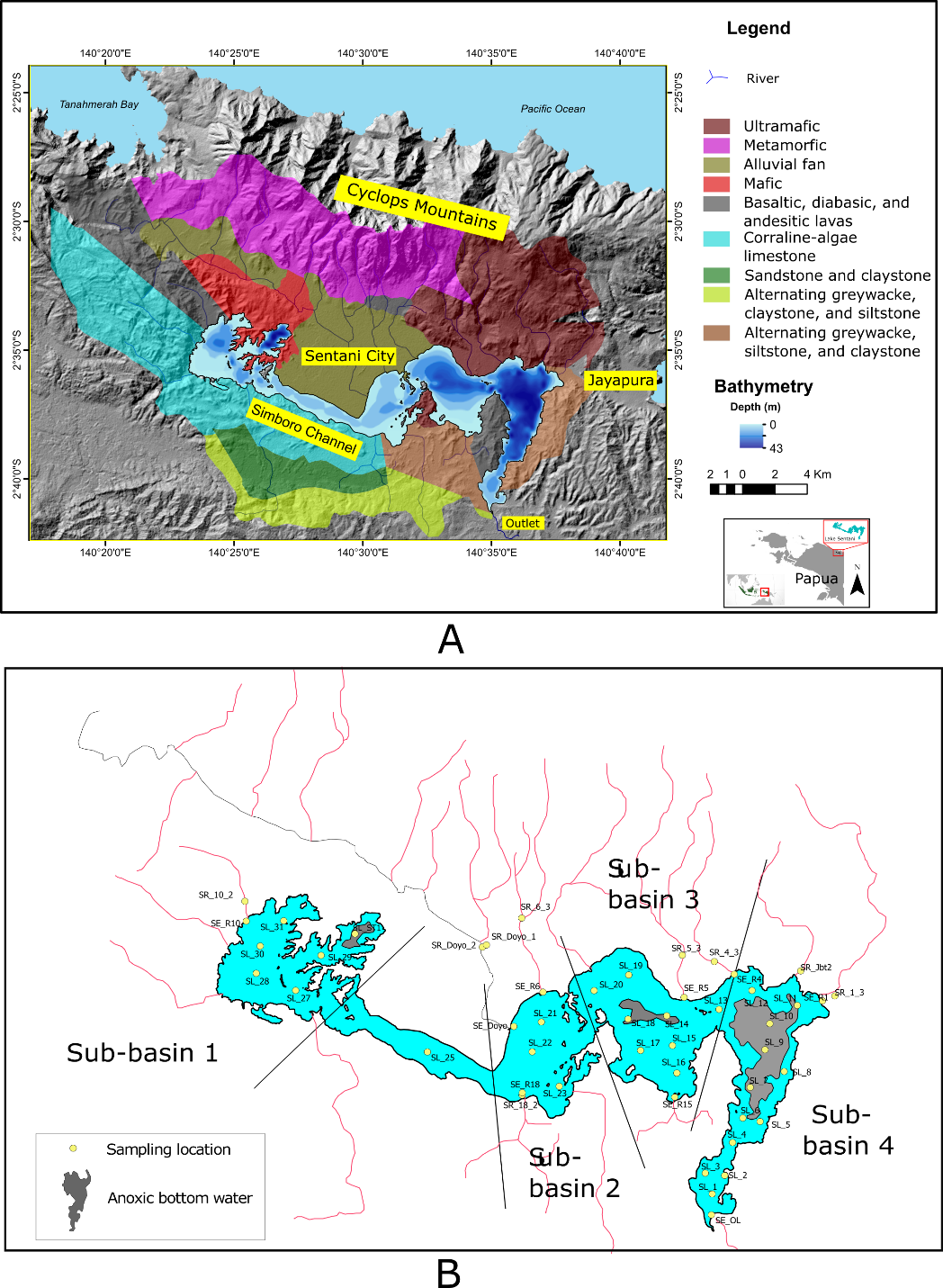

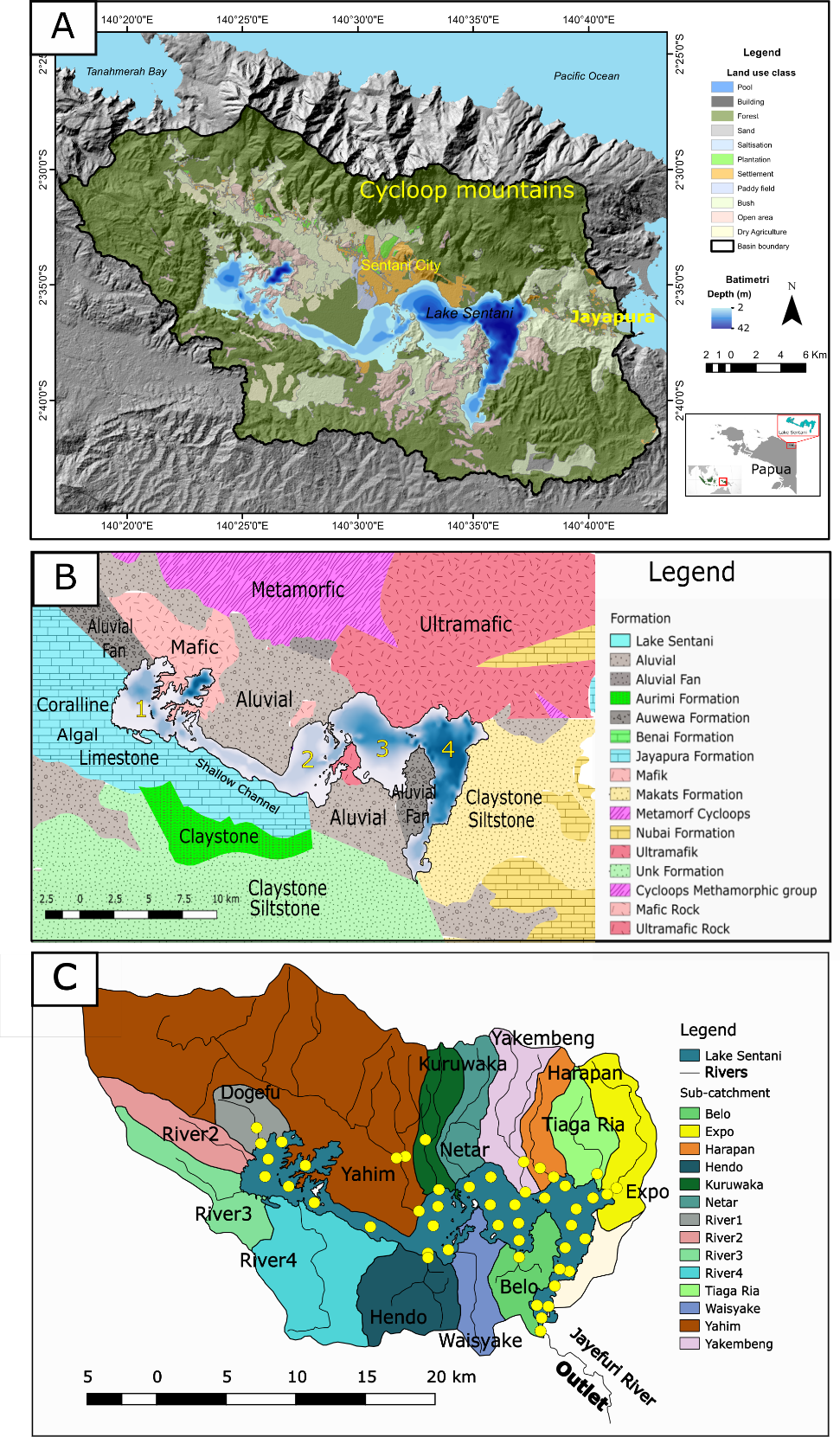


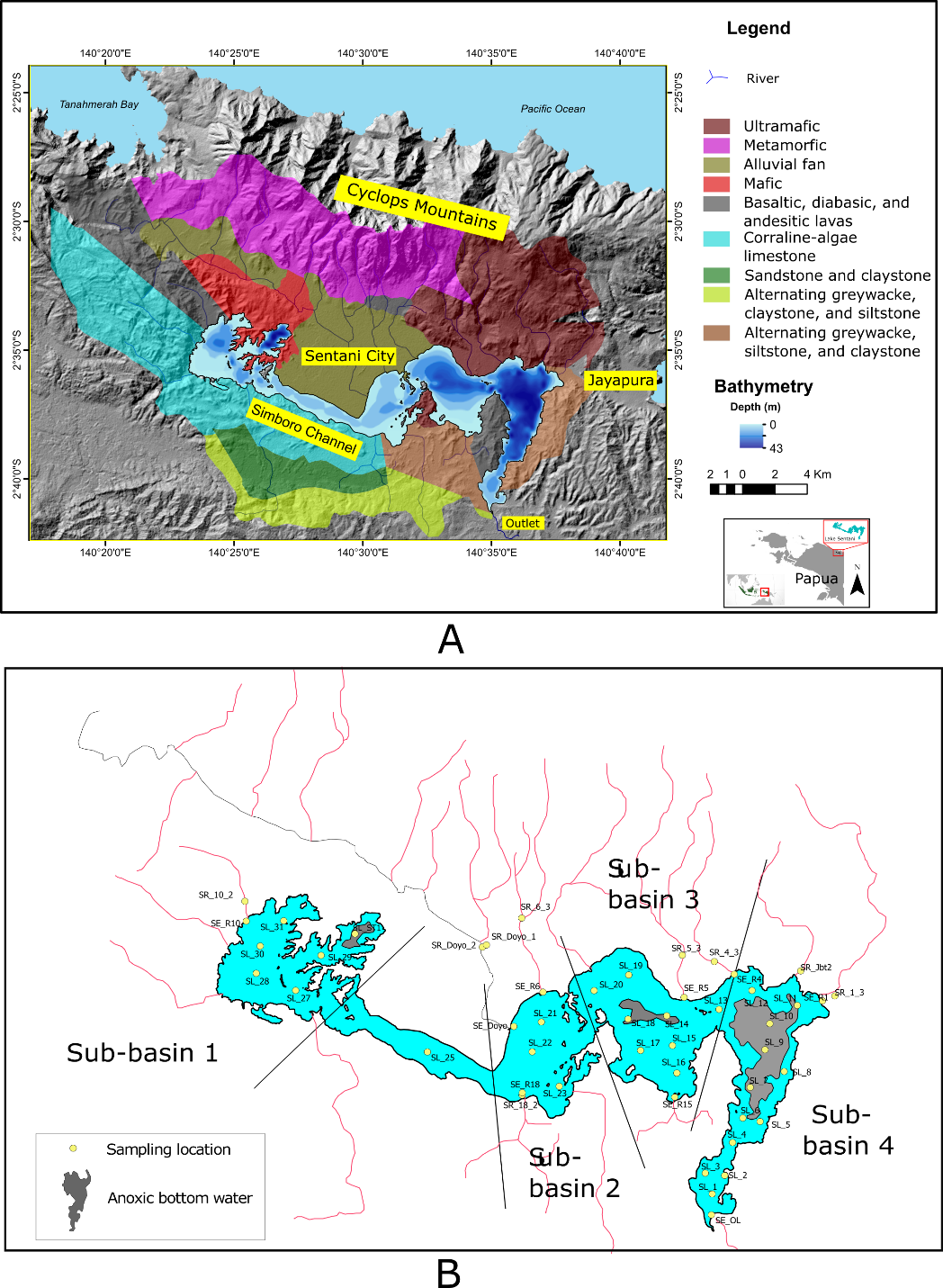


**C**

**Figure SI 1.** Lake Characteristics. (A) Map of Lake Sentani and its surrounding watershed. Catchment lithology is indicated by different colors. The lake is bounded by the Cyclops Mountains to the north and lowlands to the south. The low-resolution bathymetric map is modified after Sadi (2014) with the deepest part at 43 m, the lithology of Lake Sentani’s catchment is modified after Suwarna and Noya (1995) and the catchment boundary is modified after Sartimbul et al. (2015). (B) The Sub-catchments in Lake Sentani (modified after Sartimbul et al., 2015) and sampling location (yellow circles). (C) The division of sub-basins and anoxic bottom water zones in Lake Sentani.

**Figure SI 2**. Alpha diversity measurements based on the relative abundance of observed ASVs: A) Species richness (S), B) Shannon indices (H), and C) Evenness indices (J). The habitats refer to Figure 1B. Before calculation, each sample was rarefied 100 times to a depth of 10,000 sequences. P=values are depicted at top of each figure.

**A**


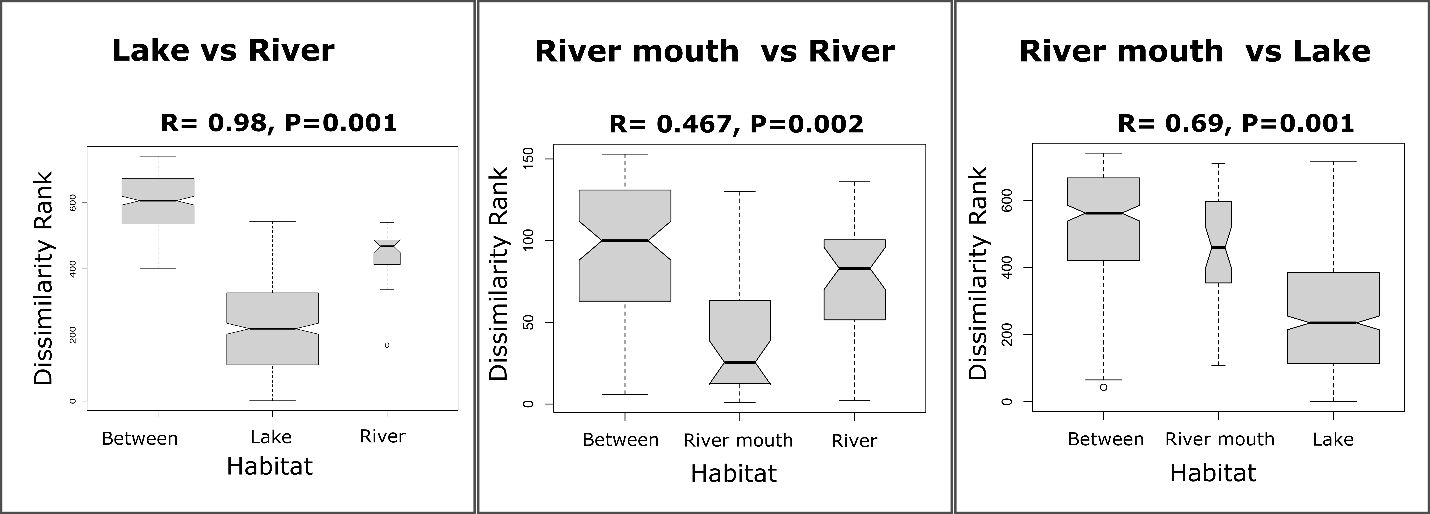


**B**

**
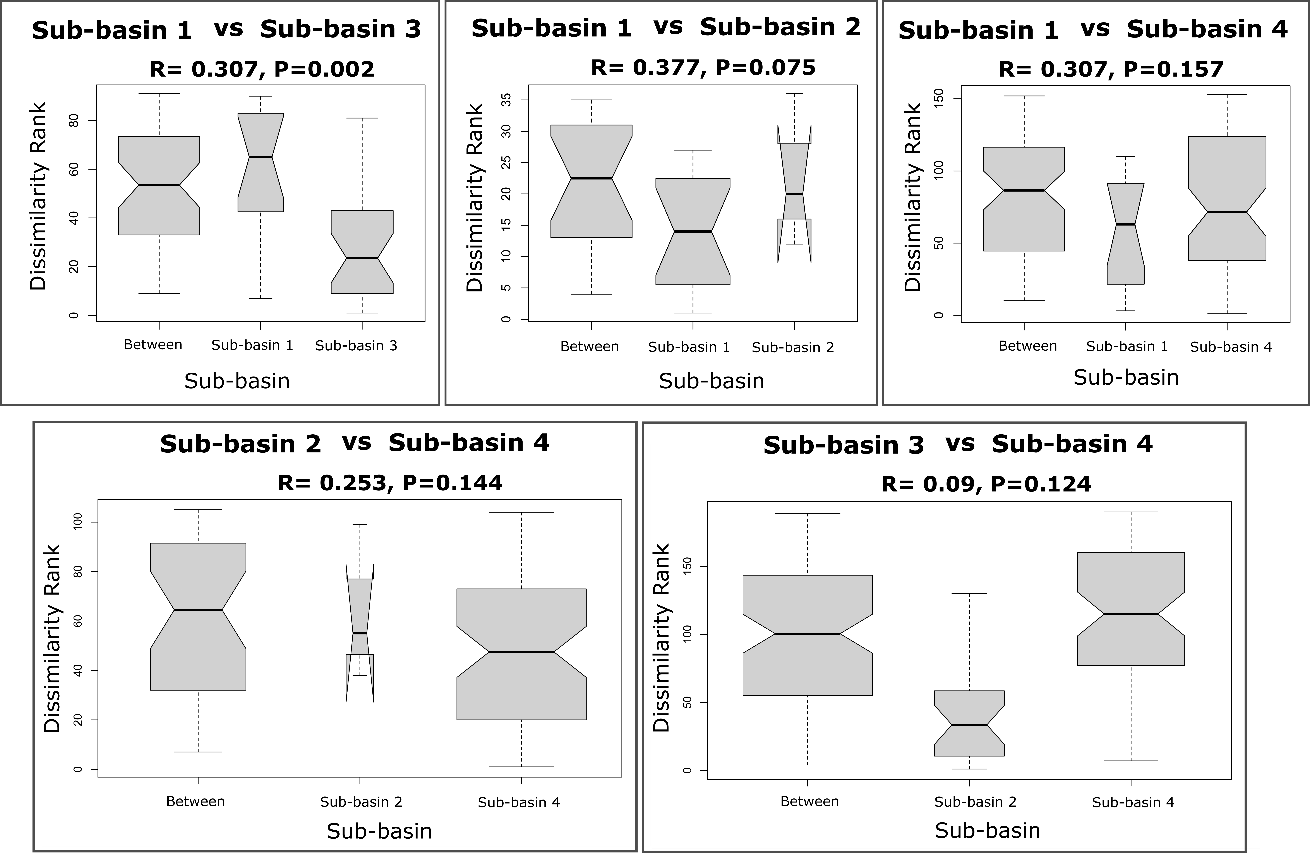
**

**
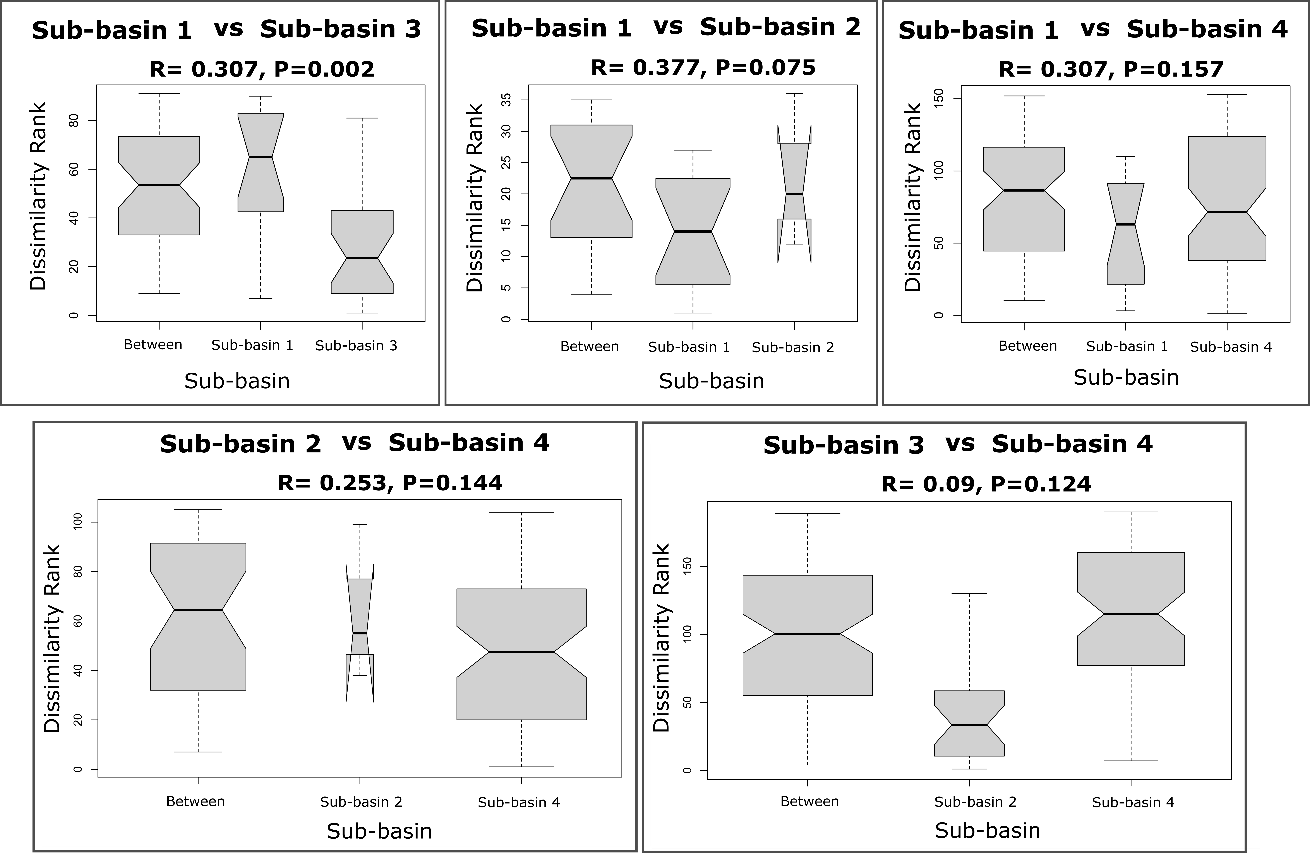
**

**C**

**
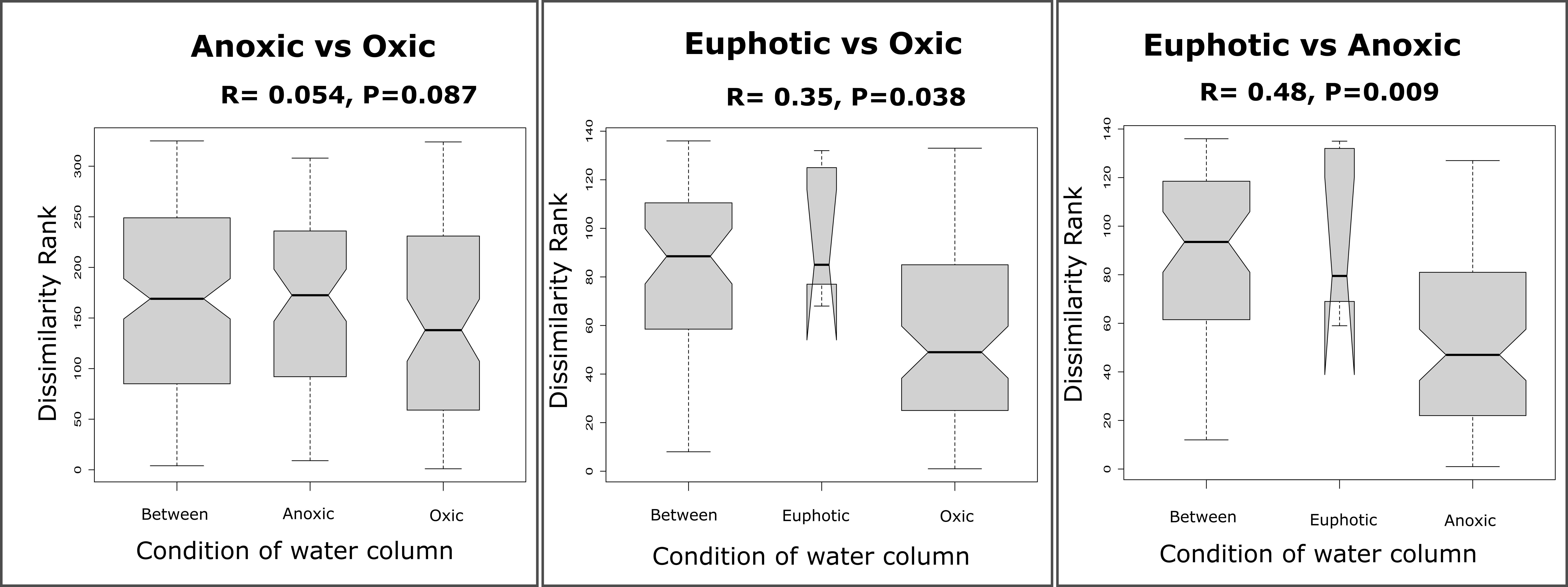
**

**Figure SI 3.** ANOSIM (Analysis of similarity) test results between habitats (A), sub-basins (B) and the condition of water column (C). R-value indicates a very high degree of separation between habitats which ranges from -1 to 1, with 1 indicating complete separation of habitats and -1 indicating complete overlap. Significance if *p* <0.05. Permutation 999. The dissimilarity metric used is Bray-Curtis dissimilarity, which measures the difference in species composition between habitats.


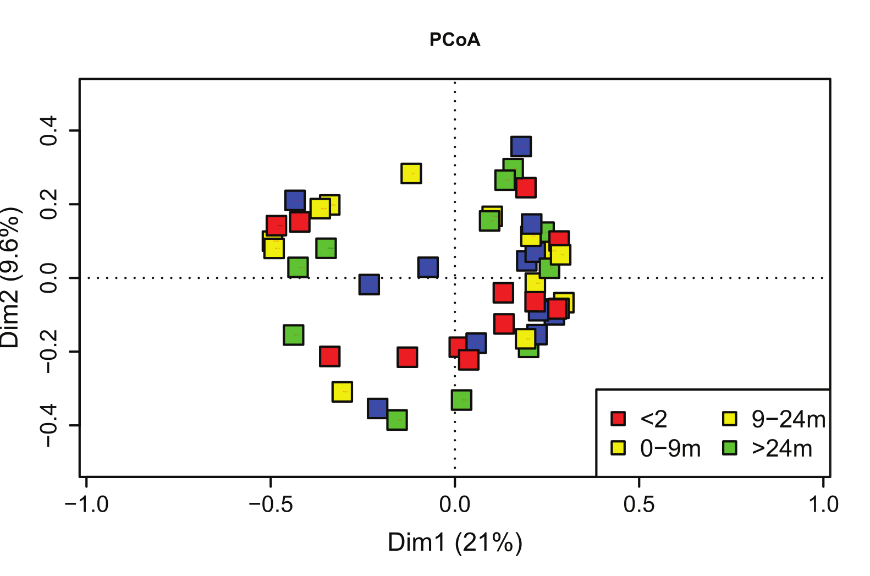


**Figure SI 4.** Microbial Community Structure Analysis of Euphotic (<9 m) and Anoxic Zones (>24m) using PCoA and Bray-Curtis Distances.


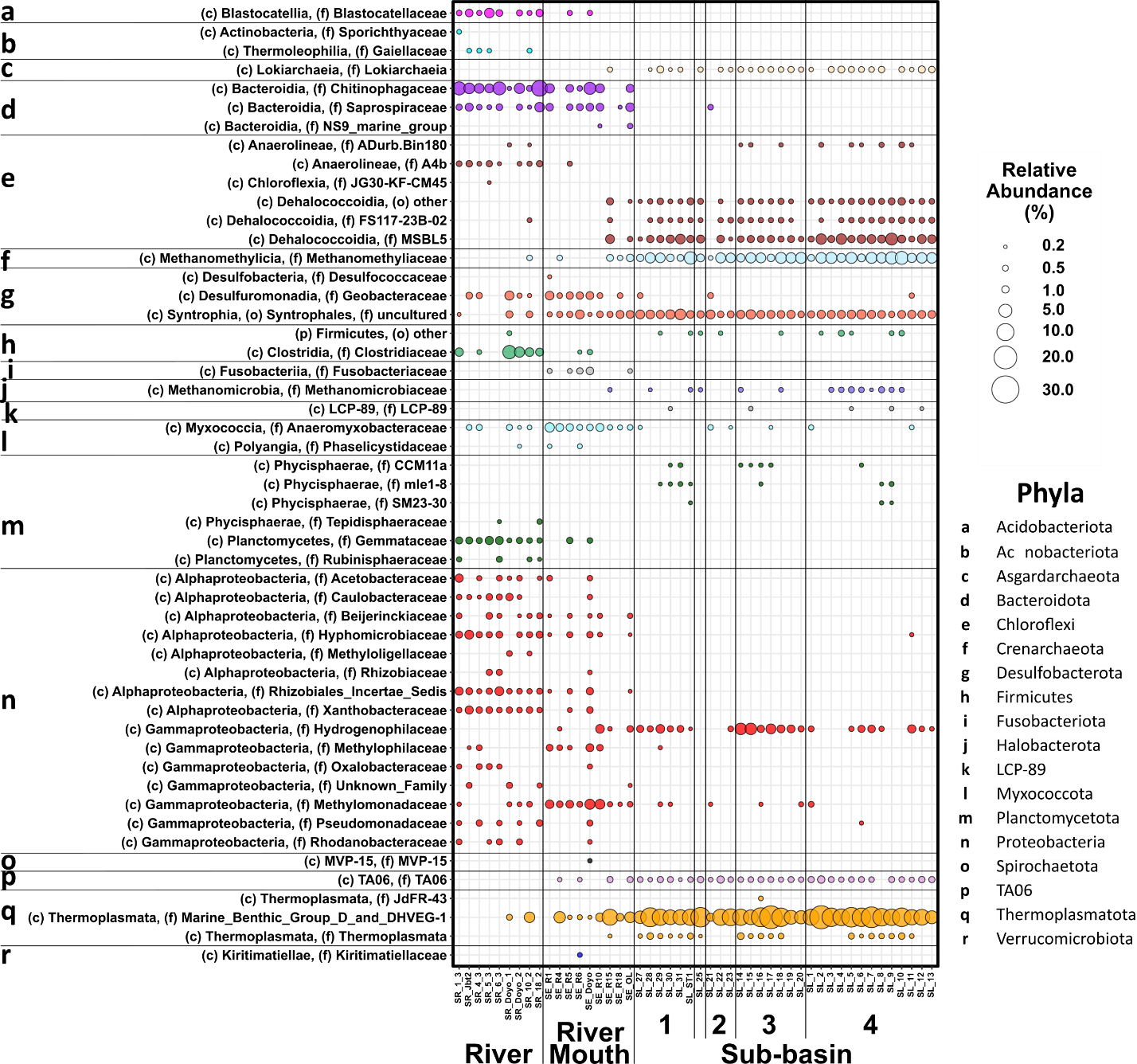


**Figure SI 5**. Bubble Plot depicting microbial relative abundance at family level, as determined by Indicator Species Analysis (p=0.005), using a 0.2% abundance cut-off.


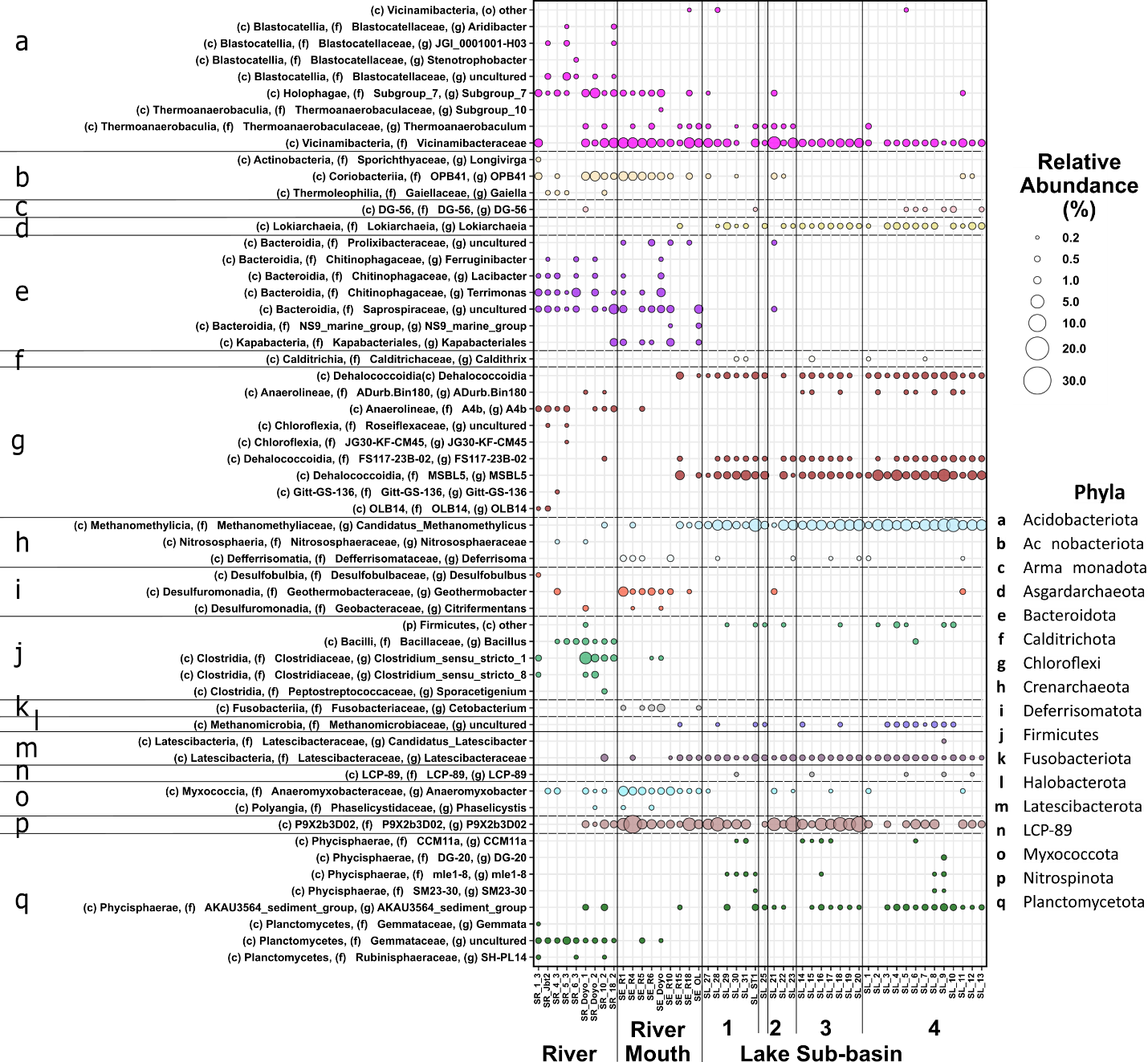


**Figure SI 6**. Bubble Plot depicting microbial relative abundance at genus level, as determined by Indicator Species Analysis (p=0.005), using a 0.2% abundance cut-off.


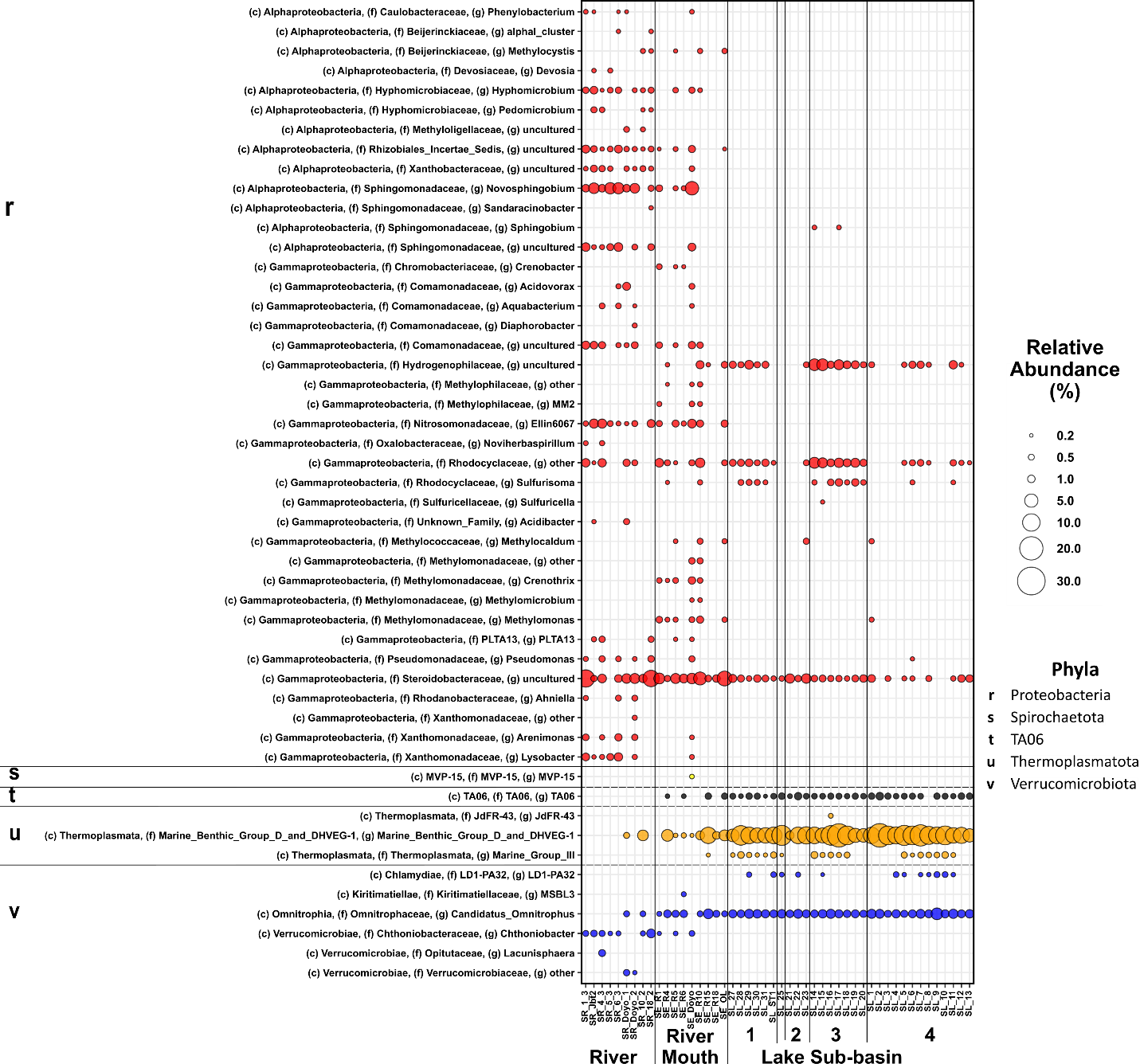


**Figure SI 6 (continued).** Bubble Plot depicting microbial relative abundance at genus level, as determined by Indicator Species Analysis (p=0.005), using a 0.2% abundance cut-off.


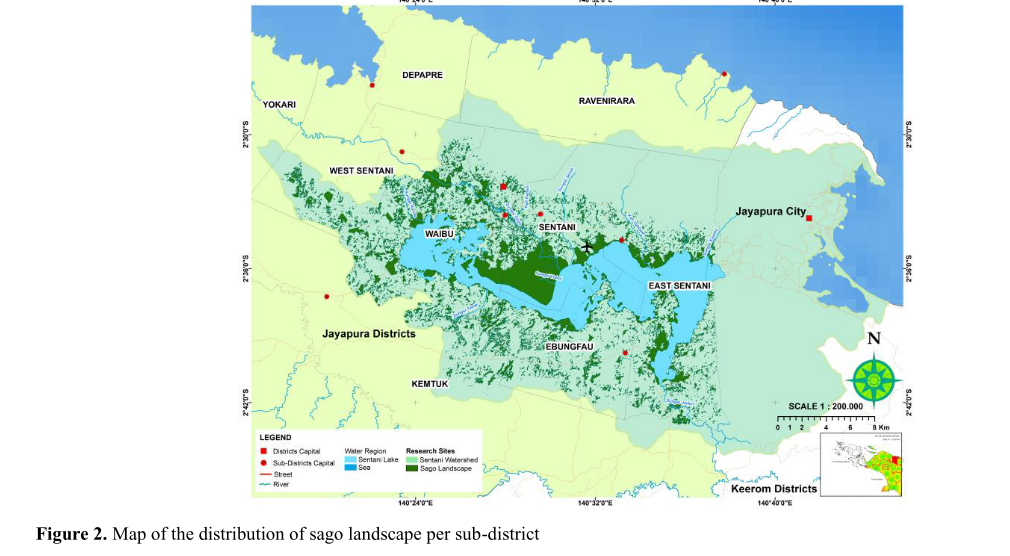


**Figure SI 7.** Map depicting Sago production along the shore of Lake Sentani. Adapted from Dimara et al, 2021


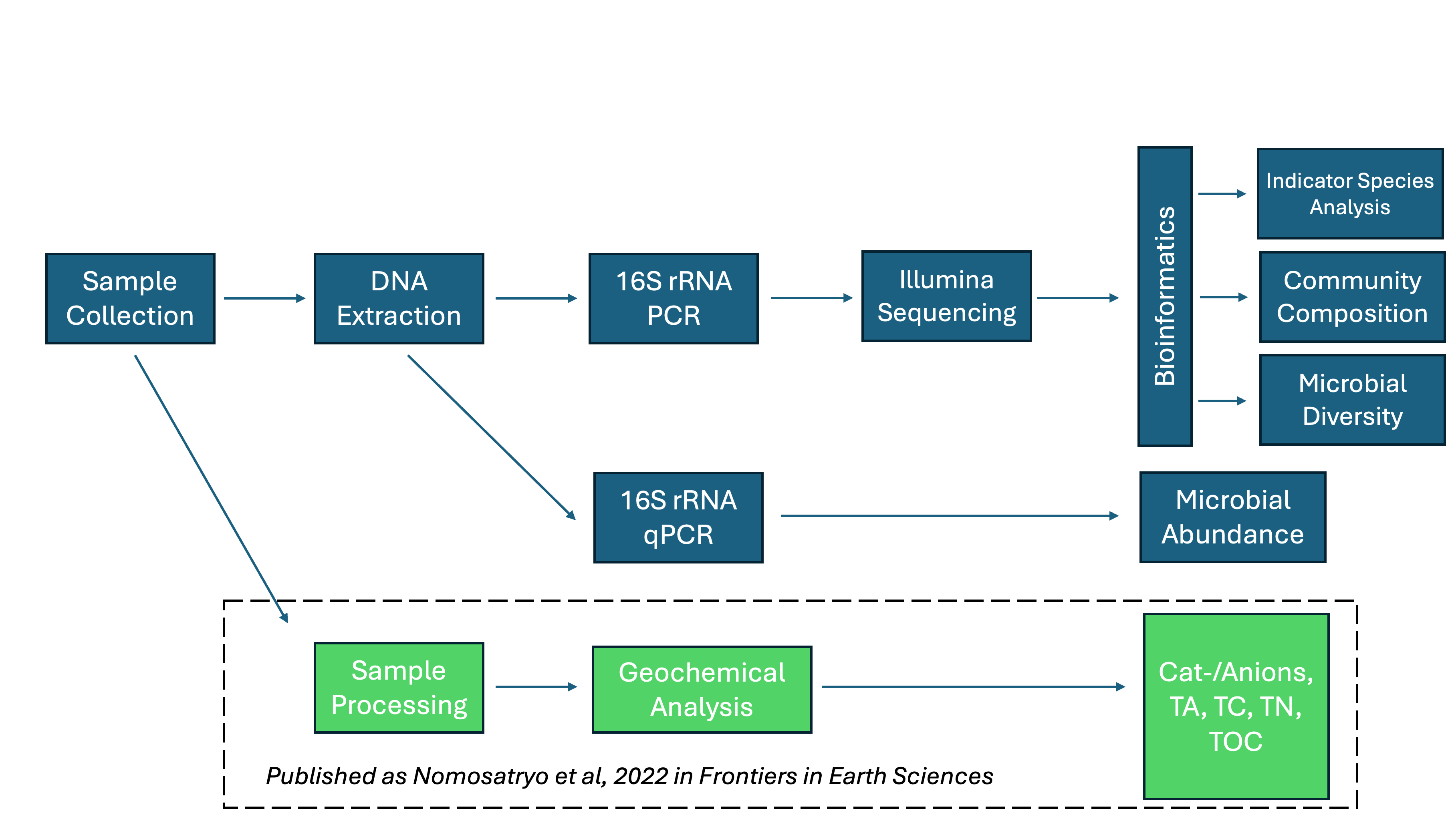


**Figure SI 8:** Flowchart of analysis workflow for this study.
